# Near-atomic structure of the inner ring of the *Saccharomyces cerevisiae* nuclear pore complex

**DOI:** 10.1038/s41422-022-00632-y

**Published:** 2022-03-18

**Authors:** Zongqiang Li, Shuaijiabin Chen, Liang Zhao, Guoqiang Huang, Xiong Pi, Shan Sun, Peiyi Wang, Sen-Fang Sui

**Affiliations:** 1 State Key Laboratory of Membrane Biology, Beijing Advanced Innovation Center for Structural Biology, Beijing Frontier Research Center for Biological Structure, School of Life Sciences, Tsinghua University, Beijing, China; 2 Department of Biology, Southern University of Science and Technology, Shenzhen, Guangdong China; 3 Cryo-EM Center, Southern University of Science and Technology, Shenzhen, Guangdong China

**Keywords:** Cryoelectron microscopy, Nuclear pore complex

## Abstract

Nuclear pore complexes (NPCs) mediate bidirectional nucleocytoplasmic transport of substances in eukaryotic cells. However, the accurate molecular arrangement of NPCs remains enigmatic owing to their huge size and highly dynamic nature. Here we determined the structure of the asymmetric unit of the inner ring (IR monomer) at 3.73 Å resolution by single-particle cryo-electron microscopy, and created an atomic model of the intact IR consisting of 192 molecules of 8 nucleoporins. In each IR monomer, the Z-shaped Nup188–Nup192 complex in the middle layer is sandwiched by two approximately parallel rhomboidal structures in the inner and outer layers, while Nup188, Nup192 and Nic96 link all subunits to constitute a relatively stable IR monomer. In contrast, the intact IR is assembled by loose and instable interactions between IR monomers. These structures, together with previously reported structural information of IR, reveal two distinct interaction modes between IR monomers and extensive flexible connections in IR assembly, providing a structural basis for the stability and malleability of IR.

## Introduction

The presence of nucleus, as the most distinguished hallmark between eukaryotes and prokaryotes, divides a eukaryotic cell into isolated compartments. Macromolecular transport across the two-membraned nuclear envelope (NE) is essential for spatially separated transcription and translation.^1–3^ Nuclear pore complexes (NPCs), embedded in NE, are the massive bidirectional transport channels between the nucleus and the cytoplasm.^4^ Since the first glimpse about seventy years ago,^5,6^ NPCs have attracted increasing attentions from researchers. The NPC is the largest proteinaceous assembly in vivo, which consists of ~550–1000 molecules of ~30 different nucleoporins (Nups) with the molecular mass of 60–120 MDa from yeast to human, and are the sole gatekeepers controlling the nucleocytoplasmic transport of macromolecules.^7–9^ Perturbations of NPCs are known to cause or promote numerous diseases, including viral infections, developmental disorders, neurodegenerative diseases and cancers.^10–14^

It has always been a challenging task to comprehensively understand the elaborate structure and formidable function of the NPC giving its huge size, complexity, flexibility and highly dynamic nature.^15,16^ In recent years, profiting from the innovation of cryo-electron microscopy (cryo-EM) technology and the development of protein purification method, several teams have acquired the intact NPC samples successfully and determined the three-dimensional (3D) maps at 20–30 Å resolutions, including *Saccharomyces cerevisiae* NPC (*sc*NPC),^17,18^ *Chlamydomonas reinhardtii* NPC (*cr*NPC),^19^ *Xenopus laevis* NPC (*xl*NPC)^20–22^ and *Homo sapiens* NPC (*hs*NPC).^23,24^ However, owing to the barrier of low resolutions, the accurate molecular arrangement of NPC subunits remains thus far enigmatic.

Earlier analyses of protein–protein interactions and mass spectroscopy (MS) studies suggested that the NPC can be divided into several stable subcomplexes, including the well-established Y complex,^25–27^ the scaffold inner ring complex,^24,28^ the channel nup trimer (CNT) complex,^28,29^ cytoplasmic 82 complex,^30,31^ nucleoplasmic basket complex^32^ and the transmembrane ring complex,^17,33^ which further oligomerize into a cylindrical structure. Scientists have been devoted continuously to solving the structures of these subcomplexes or subunits in an attempt to achieve the complete NPC structure as a jigsaw puzzle.^24,25,28,29,31^ However, only some fragments of subunits and partial structures of minor subcomplexes (except for Y complex) have been resolved directly by X-ray crystallography or cryo-EM technology so far.^21,25^ In 2018, Kim et al. published an integrated NPC model from yeast at sub-nanometer precision by satisfying a wide range of data derived from different analyses including small angle X-ray scattering (SAXS), spectroscopy, crystallography, MS, protein interactions and low-resolution cryo-EM map of the NPC.^17^ However, this model displays low accuracy for the orientation of each subunit.

As the core scaffold of the NPC, the inner ring (IR) attaches itself to the cytoplasmic, nucleoplasmic and luminal rings (CR, NR and LR), and is directly involved in the formation of the central transport channel of the NPC. Here, using single-particle cryo-EM, we report the near-atomic structures of IR, which, together with previously published structural information, reveals two distinct interaction modes between IR subunits, providing insights into the assembly mechanism of IR.

## Results and discussion

### Overall structure of the entire NPC

**Fig. 1:**
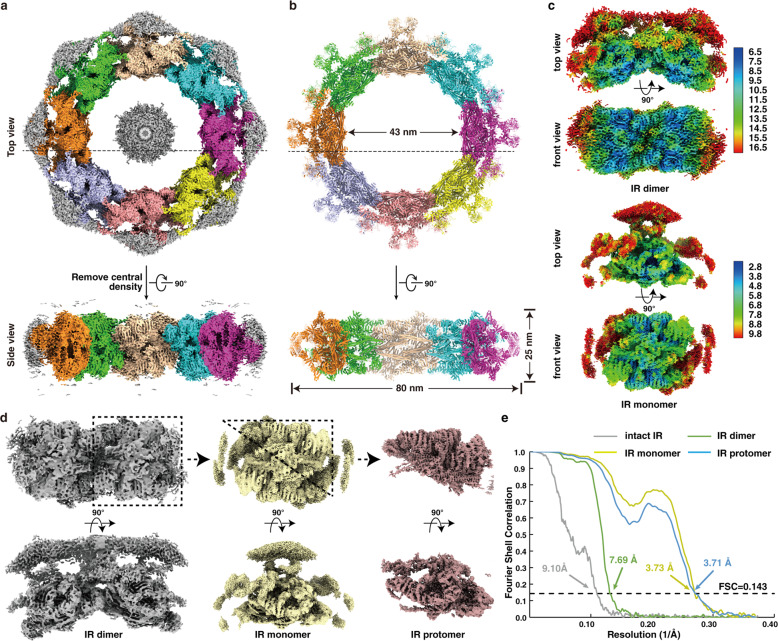
Cryo-EM structures of the IR of the *S. cerevisiae* NPC. **a**, **b** Cryo-EM density map (**a**) and the atomic model (**b**) of the intact IR. The map and model are shown in top view (upper panel, dotted lines indicate the cross section corresponding to the side view) and side view (lower panel). Different colors represent different IR monomers. Threshold contour level is 0.22 σ. The gray blob density in the middle of the IR, most probably is a certain complex of FG-repeats, nuclear transport receptors and cargoes. **c** Color-coded 3D reconstructions of IR dimer (upper panel) and IR monomer (lower panel) showing local resolutions in different views. Threshold contour levels of dimer and monomer are 0.2 σ and 0.13 σ, respectively. The local resolutions were estimated with cryoSPARC and generated in ChimeraX. **d** Density maps of IR dimer, IR monomer and IR protomer determined in this study. **e** Gold standard Fourier shell correlation (FSC) curves for the maps of intact IR, IR dimer, IR monomer and IR protomer.

Previous studies and our preliminary experiments showed great structural variability of the NPC^15,16,18,34^ (Supplementary information, Fig. S1a, c). To enrich high-quality samples of *sc*NPCs, we optimized and developed a gentle and rapid purification method referring to previously published literatures^17,35^ to increase mono-dispersity and uniformity (Supplementary information, Figs. S1b, S5b). The key step is to treat cells with a chemical reagent alpha-mating factor (α factor). α factor inhibits DNA synthesis, arrests cells in G1 phase synchronously, and makes cells present a recognizable Shmoo shape^35^ (Supplementary information, Fig. S2). Due to these effects of α factor on cells, we worried whether the structure of *sc*NPC could be changed by adding α factor. Because no relevant research had been published yet, we thus collected two datasets of NPCs extracted from yeast cells treated with or without α factor. Finally, we obtained two low-resolution structures of NPC at 24 Å resolution (+α factor, with 32,159 particles) and 26 Å resolution (–α factor, with 21,542 particles), respectively (Supplementary information, Fig. S3). Structural comparison indicated no obvious structural difference between NPCs from cells with or without α factor treatment, especially in the region of IR (Supplementary information, Fig. S3). Because the particles on the grid displayed a certain preferential orientation (Supplementary information, Fig. S1), we collected data combining the tilting angles at 0 and 40 degrees. We acquired 279,900 particles from 283,881 and 12,939 micrographs at tilting angles of 0 and 40 degrees, respectively, and finally determined the cryo-EM map of the entire NPC at ~12.0 Å using 51,220 particles by single particle analysis (SPA) (Supplementary information, Figs. S4a, S5a–d) (refer to Materials and methods for details). In agreement with previous reports,^17,18^ the resulting map of the entire NPC presents a sandwiched architecture with one layer of IR and two layers of outer rings (CR and NR) and exhibits 8-fold rotational symmetry. Besides, the controversial LR that surrounds the periphery of NPC, is visible in our map (Supplementary information, Fig. S5d). The LR was also found in tomography analysis of *xl*NPC^22^ and detergent-extracted *sc*NPC,^17^ but not in tomography of *sc*NPC,^18^ which was probably due to the limited resolution or strong noise of NE or its intrinsic flexibility. *sc*NPC spans 60 nm along the nucleocytoplasmic axis, and ~100 nm parallelly to the equatorial plane of NPC (Supplementary information, Fig. S5d). In the map of the entire *sc*NPC, the quality of the IR region is better than those of CR, NR and LR (Supplementary information, Figs. S3b, S5d), which is consistent with the function of IR as the core scaffold. The IR measures 80 nm and 43 nm in outer and inner diameters, respectively, which are the same as those published by Seung JK et al. and narrower than its in-cell diameter (Fig. 1a, b; Supplementary information, Fig. S6a).^17,18^ Although the diameters of the entire IR vary even within the same species or across different cellular states,^17,19,36^ the asymmetric element of IR, here namely IR monomer, is very similar in structural characteristics even among different species (Supplementary information, Fig. S6b), suggesting that the IR monomer is relatively conserved and rigid in the variable IR.

### Near-atomic structure of IR at 3.73 Å

Next, we tried to improve the resolution of nodical IR region from the entire NPC map. Firstly, we extracted one IR monomer of each NPC particle from raw micrographs based on the angular parameters of the intact NPC and the relative position between IR monomer and the NPC. Then we refined the contrast transfer function (CTF) and angular parameters of the extracted IR monomers. Finally, the optimized parameters were applied back to the reconstruction of the entire IR (refer to Materials and methods for details). By using this strategy, we obtained a final density map of the entire IR at 9.10 Å resolution (Fig. 1a, e; Supplementary information, Figs. S4b, S5e), which appears to be shaped like a “Finger Ring” (Fig. 1a). In order to further improve the resolution of the IR monomer, we extracted each IR monomer and combined them for further processing (Supplementary information, Figs. S7, S8a). The overall resolution of the resulting IR monomer is 3.73 Å with local resolutions of 2.8–3.4 Å at the interaction region between Nup188 and Nup192 (Fig. 1c, e), where side chains are clearly visible for most residues (Supplementary information, Fig. S9a, b). In order to improve the resolution to aid model building, we performed symmetry expansion of each IR monomer particle, which resulted in 2-fold particles of IR protomer (Supplementary information, Fig. S8a) (refer to Materials and methods for details). Although the final resolution of the IR protomer is 3.71 Å (Fig. 1d, e; Supplementary information, Fig. S7e), only slightly higher than that of the IR monomer, the map quality of CNT is much better than that in the IR monomer.

Since the density map of the monomer is of poor quality at the peripheral region, we then performed reconstruction of the IR dimer to improve map quality at the connection region between the IR monomers (Supplementary information, Fig. S8b). A reconstruction of IR dimer was obtained at 7.69 Å resolution (Fig. 1d, e), in which Nup170 dimer and Nic96 molecules can be clearly recognized (Fig. 1c; Supplementary information, Fig. S9c).

The much-improved quality of IR monomer enabled us to place IR subunits into correct orientations more accurately than before. Special S-shaped Nup188 and Nup192 are easily identified in the map with local near-atomic resolution, and the representative long stalked α-helices from CNT complexes close to Nup188 and Nup192 are also conspicuous (Fig. 1d; Supplementary information, Fig. S7e). Many α-helices of the α-solenoid domains from Nup170 and Nic96-B-1/2 are seamlessly fitted into the tubular densities that are characteristic of α-helices (Supplementary information, Fig. S9a). Although the peripheral density containing Nup157 and Nic96-A-1/2 exhibits poor quality, we recognized them using previously reported integrative models,^17,18^ and the final models of Nup157 and Nic96-A-1/2 fit well into the corresponding EM maps of IR monomer and IR dimer, with cross-correlation coefficients at 0.83 and 0.84, respectively.

We finally generated an atomic model of IR monomer, which is composed of two copies of large subunits, Nup188, Nup192, Nup157 and Nup170, and four copies of Nic96 and the CNT complex (including Nup57, Nup49 and Nsp1) (Supplementary information, Figs. S10, S11a). Eight copies of IR monomer were docked into the above entire IR reconstruction to generate a near-atomic model of IR, which includes 192 subunits and account for ~ 1/3 mass of the entire NPC with ~16 MDa (Fig. 1b; Supplementary information, Figs. S10, S11a). The orientation of almost all IR subunits in our high-resolution structure is different from that in the previously published integrated IR model (Supplementary information, Fig. S11b).

### Molecular architecture of the full-length Nup188

**Fig. 2:**
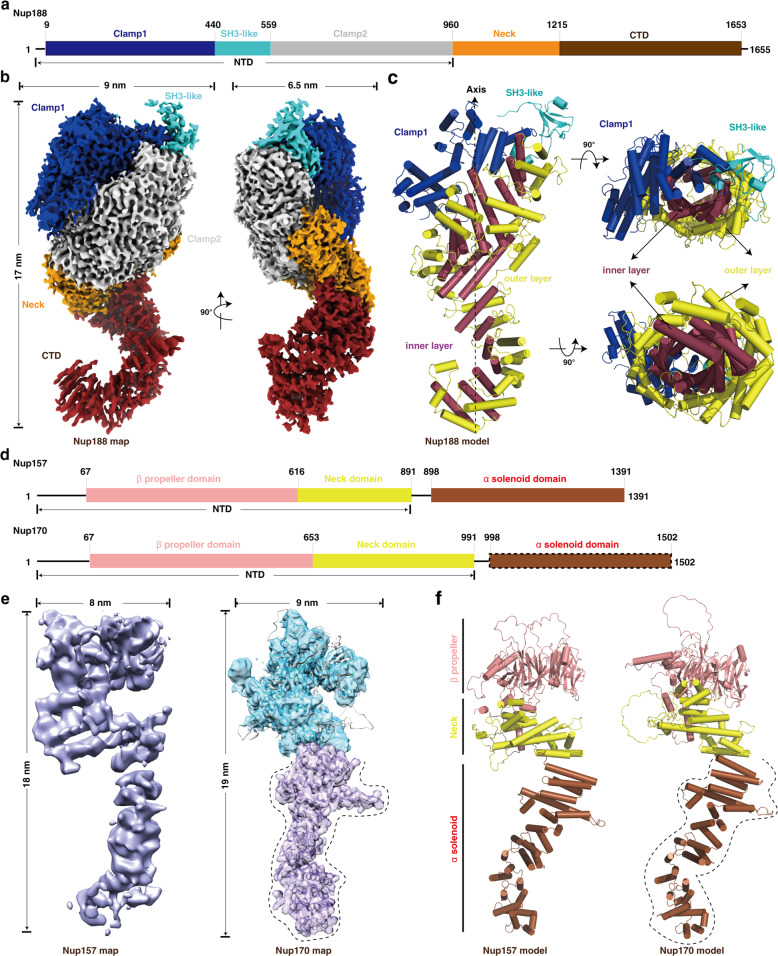
Structures of the purified full-length Nup188, Nup157 and Nup170 proteins. **a** Schematic representation of the domain structures of Nup188. Domains are color coded. **b** Final cryo-EM map and dimensions of Nup188. The contour level is 3.95 σ. Colors of domains are the same as in **a**. **c** Model of Nup188. Colors of Clamp1 and SH3-like domains are the same as in **a**, and the “S” domain is divided into two layers ― inner layer and outer layer, colored in raspberry and yellow, respectively. **d** Schematic representation of the domain structures of Nup157 and Nup170. Domains are color coded. The α-solenoid domain (boxed by dashed line) in Nup170 indicates missing regions in our map of Nup170. **e**, **f** Final cryo-EM maps (**e**) and models (**f**) of Nup157 and Nup170, respectively. The map of Nup170 is transparent and overlapped with its model. Colors of domains and dotted line marked regions are the same as in **d**. Cylinders represent α-helices. NTD, N-terminal domain; CTD, C-terminal domain.

Nup188 and Nup192, two of the largest subunits in the NPC, are evolutionary conserved homologous nucleoporins. Since the density maps of Nup188 and Nup192 are not completely resolved in the IR monomer map, we next performed SPA of the purified Nup188 and Nup192 samples, respectively, and successfully obtained the full-length structure of Nup188 at a resolution of 2.81 Å (Supplementary information, Fig. S12). Overall Nup188 appears to be a cray shape with a dimension of ∼170 × 90 × 65 Å (Fig. 2b). The N-terminal part contains two “crab claw” domains, clamp1 and clamp2, and between them is the predicted conserved SH3-like domain. The Neck domain is at the corner followed by a crescent C-terminal domain (CTD) (Fig. 2a, b). The relatively poor density of the SH3-like domain and the tail of CTD represent intrinsic flexibility of these regions (Supplementary information, Videos S1 and S2), which is consistent with their roles in mediating interactions with neighboring IR monomers (Fig. 7f). The clamp2, Neck and CTD domains further form a large right-handed super-helical ring that resembles the letter “S” comprising 52 stacked helices (Fig. 2c; Supplementary information, Fig. S13a). Surrounding the axis of the “S”, two layers of concentric α-helices spiral up in parallel and are referred to as the outer/inner layer with respect to their distances to the axis, which probably enables Nup188, like a spring, to stretch and shrink to a certain extent (Fig. 2c). The Nup188 map obtained by SPA (Nup188_SPA_) fits into the IR monomer map very well and has a good match with the Nup188 region of the entire IR monomer map (Nup188_IR_), which shows that the obtained IR monomer map by SPA has high precision and accuracy (Supplementary information, Fig. S14a).

Due to aggregation and a modest yield in both prokaryotic and eukaryotic expressing systems, we failed to resolve the structure of Nup192 by SPA. We generated the homologous model of Nup192 based on the structure of *ct*Nup192 (*Chaetomium thermophilum* Nup192), which is similar to the Nup188 model (Supplementary information, Figs. S13b, S14b). Compared with Nup188, Nup192 has an opening between clamp1 and clamp2 and a shorter tail in CTD. Another notable feature of Nup192 is the longer tower helix near the CTD, which is shorter in Nup188 and clearly visible in the Nup192 map derived from the entire IR monomer map (Supplementary information, Fig. S14b, c). Moreover, the feature of Nup188 distinguishing it from Nup192 is the existence of the N-terminal SH3-like domain, which is composed of several β-strands and loops (Fig. 2c; Supplementary information, Fig. S14c).^37^ The overall S-shaped conformation shared by Nup188 and Nup192 may play important roles in regulating and maintaining the malleability of IR.

### Molecular architectures of the full-length Nup157 and Nup170

Nup157 and Nup170 are two homologous nucleoporins. Previous studies have reported that the N-terminal membrane-binding motifs (MBMs) of Nup157 are required for membrane binding and crucial for NPC assembly,^23,38^ suggesting that Nup157 and Nup170 might be helpful in anchoring IR to NE. From the density maps of IR monomer and IR dimer (Fig. 1; Supplementary information, Fig. S15), we found that the N-terminal ends of Nup157 and Nup170 protrude into the NE, indicating that the N-terminal domains of Nup157 and Nup170 participate in the conjunction between IR and LR. To further gain insight into the structures of Nup157 and Nup170, we performed SPA of the purified Nup157 and Nup170 samples, respectively. The structures of the full-length Nup157 and the N-terminal part of Nup170 were resolved at resolutions of 5.9 Å and 4.09 Å, respectively (Supplementary information, Figs. S13c, d, S16, S17). Integrative models of the full-length Nup157 and Nup170 were produced by combining the SPA results with the previously reported X-ray structures of the N-terminal fragment of Nup157 (PDB: 4MHC) and the C-terminal part of Nup170 (PDB: 3I5P) as well as the maps of IR monomer and IR dimer obtained in this study. Nup157 and Nup170 are structurally spoon-shaped and composed of three distinct regions, a compact β-propeller domain followed by a short-stalked Neck domain and a long super-helical α-solenoid domain. The β-propeller domain and the Neck domain further form a U-shaped N-terminal domain (Fig. 2d–f). The full-length Nup157 has dimensions of ~180 Å in height and 80 Å in width. Structural analysis suggested that Nup157 is intrinsically flexible, especially in the α-solenoid domain (Supplementary information, Fig. S16g and Video S3), which might suggest its versatile role in regulating IR and LR. Different from the previously reported C-shaped negative stain structure,^39^ Nup170 shows a similar structure with Nup157, but with slightly larger dimensions, ~190 Å in height and 90 Å in width (Fig. 2e, f), which is reminiscent of its possible synergistic function with Nup157. Comparing the NTDs of Nup157 and Nup170, one obvious difference is that there are two protruding horns at the bottom of the U-shaped structure of Nup170 (Supplementary information, Fig. S17h). Easy degradability of Nup170 during purification suggests that its C-terminal domain is more flexible than that of Nup157 (Supplementary information, Fig. S17a). The flexibility of Nup157 and Nup170 might reflect their functional roles in the regulation of assembly and variability of NPC.

### Extensive interactions within IR monomer

**Fig. 3:**
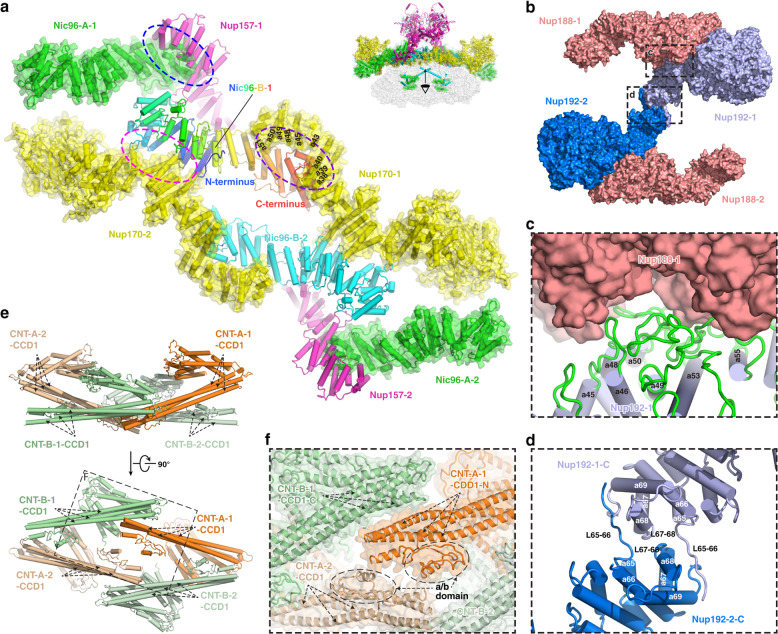
Intra-layer interactions in the IR monomer. **a** Interactions within the outer layer. Purple, magenta and blue dashed ellipses represent interactions between Nic96-B-1 and Nup170-1, Nic96-B-1 and Nup170-2, and Nic96-A-1 and Nup157-1, respectively. **b** Interactions within the middle layer. Two interfaces are boxed by black dashed line and enlarged in **c** and **d**, respectively. **c** Details of interaction between Nup188-1 and Nup192-1. **d** Details of the interaction between two Nup192 proteins. **e** Interactions within the inner layer. The black dashed box is enlarged in **f**. **f** Details of the interaction between CNT complexes.

Based on the near-atomic model of IR monomer, we analyzed the interactions between the subunits. The IR monomer can be divided into three layers (Supplementary information, Fig. S10). The outer layer, shaped like a triangular rooftop from the top view, is comprised of two copies of Nup157 (Nup157-1/2), two copies of Nup170 (Nup170-1/2) and four copies of Nic96 (Nic96-A-1/2, Nic96-B-1/2) (Fig. 3a; Supplementary information, Fig. S10 and Video S4). The middle layer contains two copies of Nup188 and two copies of Nup192, which assembles into a pore-facing Z-shaped pattern with an arched cavity on the cross section. The inner layer includes four CNT complexes (CNT-A-1/2, CNT-B-1/2) and each CNT complex is composed of three homologous proteins, Nup57, Nup49 and Nsp1 (Supplementary information, Fig. S10 and Video S4). In our model, the four CNT complexes assemble into a rhombic tetramer residing in the arched cavity of the middle layer (Fig. 3e; Supplementary information, Fig. S10), which is directly involved in the formation of the central transport channel of the NPC (Fig. 1b; Supplementary information, Fig. S10).

Within the outer layer, the N-terminal ends of Nup157-1/2 and Nup170-1/2 extend into the LR (Supplementary information, Figs. S10, S15). The C-terminal regions of Nup170-1/2, together with four diagonally arranged Nic96, form a slightly curved plane facing towards the pore of NPC, just like a bridge deck. Nic96 plays a key role in this assembly. The C-terminus of each Nic96-B is embedded in a groove formed by the helices of α38–α40, α43, α45 and α48–α51 from the C-terminal α-solenoid domain of Nup170 (Fig. 3a, purple circle; Supplementary information, Fig. S13d, e). Meanwhile, its N-terminus directly contacts the N-terminal region of α-solenoid domain of the other Nup170 via α-helices and loops (Fig. 3a, magenta circle). By this way, two Nic96 molecules (Nic96-B-1/2) and two Nup170 molecules (Nup170-1/2) are linked together and form a rhomboidal plane, which is the center of the bridge deck. Nup157-1/2 is anchored to this bridge by association of its C-terminus with the C-terminus of Nic96-A-1/2 (Fig. 3a, blue circle; Supplementary information, Fig. S13c, e), consistent with the results of cross-linking MS.^17^

For the middle layer, benefiting from the full-length structure of Nup188 and the improved resolution of IR monomer map, we found an extensive compact interface between Nup188 and Nup192, with ~2157 Å^2^ buried surface area (Fig. 3b, c), proving a direct interaction between the two large subunits, which is distinct from the in vitro results.^40^ The interaction interface is mainly composed of flexible loops and small α-helices between residues ~174–819 of Nup188 and residues ~1060–1432 of Nup192 (Fig. 3c; Supplementary information, Fig. S13a, b). Moreover, two question marker-shaped Nup192 molecules dimerize into an arch shape with an interface area of ~968 Å^2^; the interface is formed by the flexible C-terminal α-helices α65–α69 and loops L65–L66 and L67–L68 from each Nup192 (Fig. 3d; Supplementary information, Fig. S13b).

The three homologous proteins (Nsp1, Nup49 and Nup57) forming the CNT complex in the inner layer are structurally featured by the canonical heterotrimeric coiled-coil domains (CCD1, 2 and 3) for all proteins and an additional α/β domain for Nup57^28^ (Supplementary information, Fig. S13f–h). Linkages between the four CNT heterotrimers are primarily through the CCD1. In detail, the N-terminal fragment of CCD1 in CNT-A-1 is adjacent to the C-terminus of CCD1 in CNT-B-1, and simultaneously, the tips of CCD1 including the α/β domain from CNT-A-1 and CNT-A-2 are closely associated (Fig. 3e, f). In the rhombic tetramer each CNT monomer contributes a long cylindrical CCD1 domain, which is situated in the innermost part of the inner layer (Supplementary information, Fig. S10). The N-terminal part of CCD1 has been identified as the place where the extremely flexible Phe-Gly (FG) repeats locate.^41,42^ The absence of the FG repeat motifs in the model is likely due to their intrinsically disordered properties. The CNT rhombic tetramer has an inside room, which may provide a space for elastic deformation of the CNT complexes (Fig. 3e; Supplementary information, Fig. S10).

**Fig. 4:**
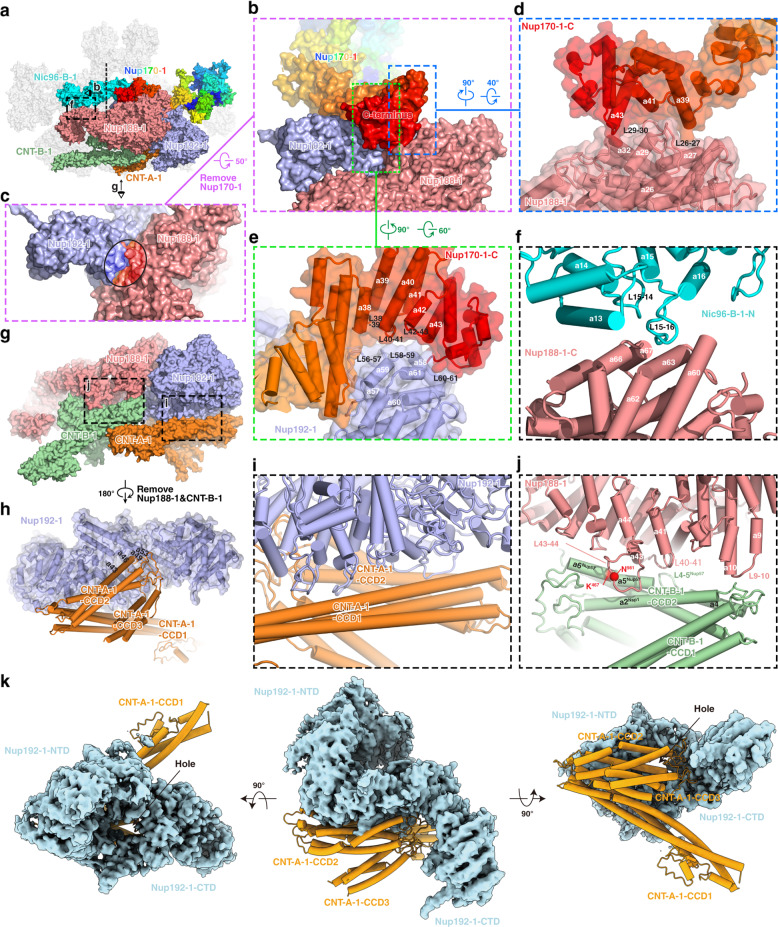
Inter-layer interactions in the IR monomer. **a** Overall view of subunits involved in the inter-layer interactions, with eye symbols and arrowheads indicating view directions shown in the following panels. **b** Interactions of Nup170-1 with Nup188-1 and Nup192-1. Two interfaces are boxed by blue and yellowgreen dashed lines and enlarged in **d** and **e**, respectively. **c** The concave surface at the junction of Nup188-1 and Nup192-1. The area contacting Nup170-1 is circled. **d**, **e** Details of the interaction of the C-terminal region of Nup170-1 with Nup188-1 (**d**) and Nup192-1 (**e**). **f** Details of the interaction between Nic96-B-1 and Nup188-1. **g** Overall view of subunits involved in the middle and inner layer interactions. Two interfaces are boxed by black dashed lines and enlarged in **i** and **j**, respectively. **h** Loops connecting CCD2 and CCD3 of CNT-A-1 protrude into the groove located in the “S” domain from Nup192-1. **i** Long stalked CCD1 and CCD2 of CNT-A-1 clamp the N-terminal corner of Nup192-1 by extensive contacts via α-helices and loops. **j** Details of the interaction between Nup188-1 and CNT-B-1. **k** CNT-A-1 blocks the hole located between NTD and CTD of Nup192-1.

A lot of interactions between different layers exist within the IR monomer (Fig. 4a). Nup170 of the outer layer makes extensive interactions with the middle layer (Fig. 4a–e). The C-terminus of Nup170 sits on the concave surface at the junction of Nup188 and Nup192 (Fig. 4b, c) and interacts with both proteins (Fig. 4d, e). It binds Nup188 by association of its α-helices α39, α41 and α43 with the α-helices α27, α29, α32 and two long loops L26–L27 and L29–L30 from the N-terminal region of Nup188 (Fig. 4d). It also interacts with Nup192 through the binding of its α-helices α38–α43 and loops L38–L39, L40–L41, L42–L43 and the C-terminal loop with α-helices α58, α59, α61, as well as loops L56–L57, L58–L59, and L60–L61 from Nup192 (Fig. 4e). Moreover, agreement with the biochemically identified interaction between Nup188 and Nic96,^17^ the α-helix α13 and the following loops L14–L15 and L15–L16 in the N-terminal region of Nic96-B-1 interact with α-helices α60, α62, α63, α66 and α67 in the C-terminal region of Nup188 (Fig. 4f). These interactions contribute to the conjunction between the outer and middle layers.

Connections between the middle and inner layers are mediated by the interaction of Nup188 and Nup192 with CNT complexes (Fig. 4g). For the interaction between Nup192 and CNT, the loops connecting CCD2 and CCD3 of CNT-A-1 protrude into the groove formed by α43, α46, α49 and α53 located in the “S” domain from Nup192-1 (Fig. 4h), and block the hole formed by the tower helix and CTD of Nup192 (Fig. 4k). In addition, the long stalked CCD1 and CCD2 of CNT-A-1 clamp the N-terminal corner of Nup192-1 by extensive contacts via α-helices and loops (Fig. 4i). There are two interaction interfaces between Nup188-1 and CNT-B-1 (Fig. 4j). One interface is contributed by the CNT surface formed by α-helix α5 and loop L4–L5 from Nup57, and α-helix α2 from Nsp1, and the Nup188 surface formed by α-helices α41 and α43, as well as loops L40–L41 and L43–L44 (Fig. 4j). This interaction has been verified by a pair of cross-linked residues identified from the endogenous *sc*NPC, Asn^981^ from Nup188 and Lys^467^ from Nup57.^17^ The other interface is formed by the loops linking CCD1 and CCD2 of CNT-B-1, and α-helix α10 and loop L9–L10 of the Nup188 N-terminus (Fig. 4j). The above multiple interactions between CNT and Nup188/192 may contribute to the propagation of conformational changes from the inner layer CNT complexes to the middle layer subunits.

**Fig. 5:**
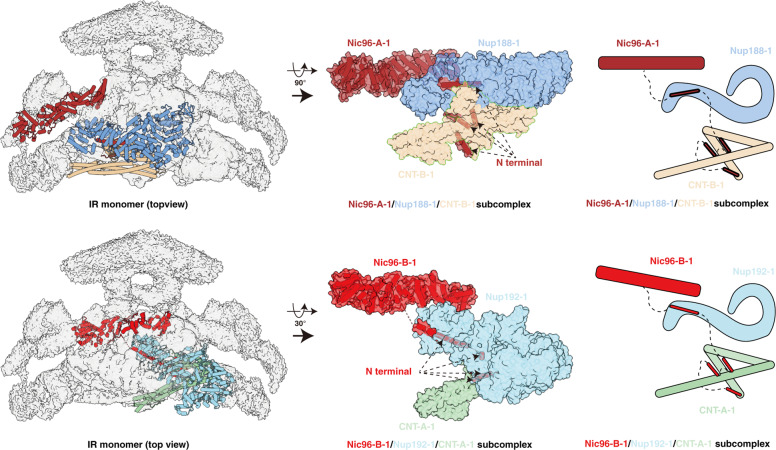
Location of N-terminal α-helices of Nic96. The N-terminal fragment of Nic96 strings three layers of IR monomer through the interaction with Nup188, Nup192 and the CNT complex by several α-helices.

In addition, several unassigned densities were found in the C-terminal regions of Nup188 and Nup192 surrounding the CCD3 of CNT. These are most likely fragments of the flexible N-terminal region of Nic96 based on the crystal structure of the Nic96–CNT complex, biochemical results and the predicted structure of the full-length Nic96^28,38,42,43^ (Fig. 5). In this case, four Nic96 molecules string all three layers of the IR monomer and interact with all IR subunits, suggesting its indispensable role as the keystone of NPC assembly (Fig. 5). Moreover, located between Nic96-A-1 and the N-terminus of Nup170, an unambiguous density might come from Nup53/Nup59 based on the MS and biochemical results (Supplementary information, Fig. S9c),^17,36^ indicating the possible function of the two flexible linker nucleoporins in the assembly of IR.

### Elastic CNT tetramer in the center of the transport channel

**Fig. 6:**
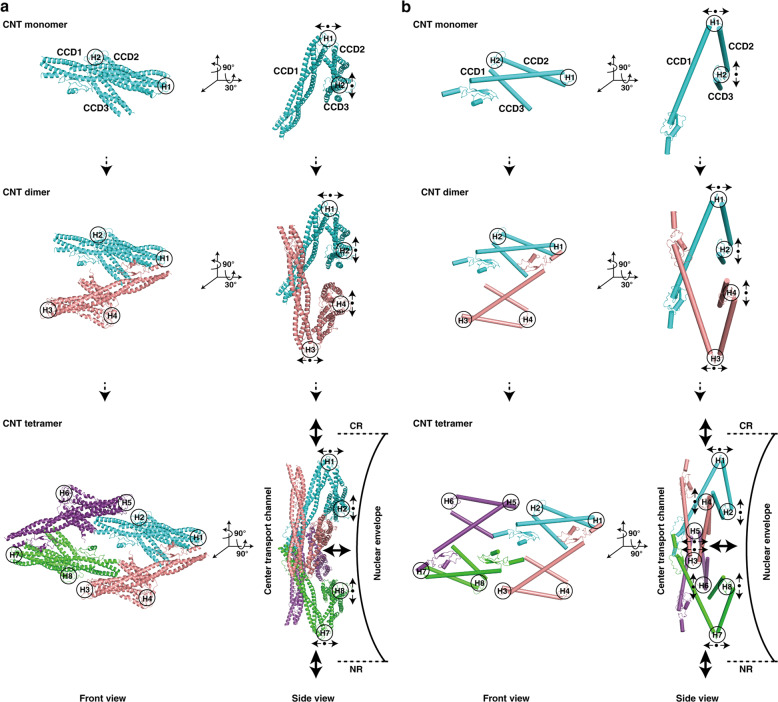
Conformational change of the CNT complexes. **a**, **b** Molecular architectures and model illustrations are shown in **a** and **b**, respectively. Top and side views are shown in left and right panels, respectively. Among three CCD domains in CNT monomer, two hinge regions (H1 and H2) regulate angular variation of adjacent CCD domains to sense cargoes with different sizes. Small black arrows represent possible motions of each CCD domain around hinge region. The resultant motions of CNT tetramer are shown by larger black arrows.

As mentioned above, four CNT complexes are arranged as a rhombic tetramer, which connects one by one to form the innermost circle of IR (Figs. 1a, b and 3e). Thus, two approximately parallel rhomboidal structures of the inner and outer layers, together with the Z-shaped Nup188–Nup192 middle layer, constitute a sandwiched construction of IR monomer (Supplementary information, Fig. S10 and Video S4). The structure of the whole IR is not very compact as demonstrated by the presence of a lot of unfilled spaces in it (Fig. 1a, b). From a mechanical point of view, the above discussed multilayer assembly and the relatively loose structure might lead to an elastic mechanism, which could mediate the transfer of the conformational changes from the inner layer to the middle and outer layers. The CNT complex in the inner layer (CNT monomer) contains three cylindrical CCD domains connected by two hinges (H1 and H2) (Fig. 6). The hinge region is unstable and prone to change. Thus, the totally eight hinge regions in the CNT tetramer could allow the entire tetramer to stretch horizontally and/or vertically (Fig. 6), making the CNT tetramer an elastic element. The innermost elements of the CNT tetramer are the long cylindrical CCD1 domains, where the FG repeats form the diffusion barrier of the central transport channel.^41,42^ The cargo may be sensed by the FG repeats, which may further induce conformational changes of CCD1 domains. Therefore, it is reasonable to deduce that the structural variability of the CNT tetramer could be an adaptation to the passage of cargoes of different sizes by own structural change.

### Molecular mechanism of dilation and constriction of NPC

**Fig. 7:**
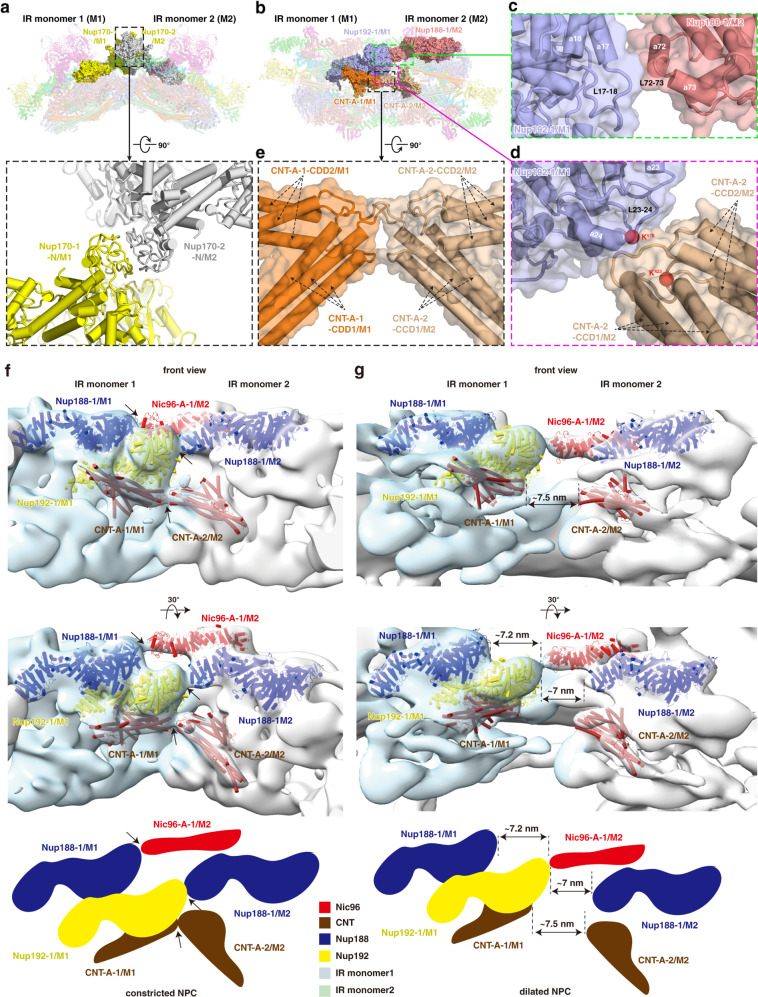
Interactions between adjacent IR monomers and comparison of dilated NPC and constricted NPC. **a** Interaction between two Nup170 molecules from adjacent IR monomers. **b** Overall view of subunits from middle and inner layers involved in the inter-monomer interactions. Three interfaces are boxed by green, magenta and black dashed lines and enlarged in **c**, **d** and **e**, respectively. **c** Details of the interaction between Nup192-1/M1 and the neighboring Nup188-1/M2. **d** Details of the interaction between Nup192-1/M1 and the neighboring CNT-A-2/M2. **e** Details of the interaction between CNT-A-1/M1 and the neighboring CNT-A-2/M2. **f**, **g** Distance changes between IR monomers are shown from constricted (**f**) to dilated (**g**) states. Two views are shown and schematic diagrams are drawn at the bottom. The representative subunits of IR are color coded. The interacting subunits of each interaction in the constricted NPC (black arrows) are separated by ~7 nm in the dilated NPC (dashed lines).

Comparison of the IR monomers in various states and from different species indicates great similarity in composition, structure and size (Supplementary information, Fig. S6). Our cryo-EM structure of the IR reveals that, as described in the above sections, three core subunits — Nup188, Nup192 and Nic96 — in each IR monomer have extensive interactions with all other subunits of the IR monomer (Figs. 4 and 5), forming a connection network to maintain the IR monomer in stable and elastic structural state. Compared with these intra-monomer interactions, the interactions between IR monomers are rather different. We found that the inter-monomer interactions are loose and instable. In the outer layer, two Nup170 molecules from the neighboring IR monomers form a herringbone conformation through the contacts of N-terminal ends via extensive interactions through loops (Fig. 7a). Besides, we also found a poor density region linking the SH3-like domain of Nup188-1/M1 and the N-terminal end of the adjacent Nic96-A-1/M2 (Fig. 7f). In the middle layer and inner layer, the α-helix α17 and loop L17–L18 of the N-terminal region of Nup192-1/M1 have a small interaction with the α-helices α72–α73 and loop L72–L73 from the flexible C-terminal region of the neighboring Nup188-1/M2 (Fig. 7b, c). In addition, the long loop L23–L24 from Nup192-1/M1 interacts with the loops connecting CCD1 and CCD2 of the neighboring CNT-A-2/M2 (Fig. 7b, d). Moreover, a binding region is formed by the hinges between CCD1 and CCD2 from both CNT-A-1/M1 and the neighboring CNT-A-2/M2 (Fig. 7b, e). All above interactions are weak and loose. Thus, our structure exhibits an interaction pattern of IR: relatively stable and strong interactions within IR monomer and relatively instable and loose interactions between IR monomers.

When we compared our IR structure with previously published integrated in situ yeast IR model,^18^ the IR diameter is significantly different: 80 nm in this study and 100 nm in the in situ study (Supplementary information, Fig. S6a). Close inspection found that the diameter difference primarily comes from the distance changes between IR monomers. In our structure, the disordered loop at the N-terminus of Nic96-A-1 interacts with the flexible SH3-like domain of the neighboring Nup188-1 (Fig. 7f), and the weak density suggests that this interaction is rather loose. While in the in situ study, Nic96-A-1 is observed to contact the adjacent Nup192-1 instead, and the other aforementioned interactions found in this study are separated by ~7 nm (Fig. 7f, g). As a result, about 90% perimeter change of IR is due to the different interaction modes between IR monomers and the resulting different distance between IR monomers. Thus, the NPCs observed in this study and in the in situ study likely present the NPCs in a constricted state and in a certain dilated state, respectively. In the constricted state, Nic96-A interacts with the neighboring Nup188, whereas in the dilated state Nic96-A changes to interact with the neighboring Nup192. Considering this together with the interactions between the four Nic96 molecules and CNT tetramers in IR monomer, we propose that the flexible N-terminal fragment of Nic96 may function as a switch to regulate the conformational change of the NPC between constriction and dilation states to adapt to the passage of various cargoes.

In summary, our cryo-EM structures describe in detail the hierarchical assembly of the *sc*NPC IR, which consists of 192 molecules of 8 Nups with an 8-fold rotational symmetry. Each IR monomer, as the unit of the entire IR assembly, displays a multilayer sandwich structure. Structural analysis reveals extensive stable interactions within IR monomers, but relatively flexible connections and two distinct interaction modes between IR monomers, providing a structural basis for the stability and malleability of IR, and a molecular mechanism underlying the conformational change of the NPC between constriction and dilation states.

## Materials and methods

### Yeast strains and plasmids

The budding yeast *S. cerevisiae* strains and plasmids used in this study are listed in Supplementary information, Table S1. All plasmids were checked by sequencing.

### Purification of the native *S. cerevisiae* NPC

The yeast strains *S. cerevisiae* (W303a and W303α) were used throughout the procedure. In the *Mlp1-PrA/Nup84-3FH* strain, endogenous *Mlp1* and *Nup84* were tagged with PrA preceded by TEV protease cleavage sequence (ENLYFQG) and 3× FLAG plus 10× His sequence, respectively, by homologous recombination of polymerase chain reaction (PCR)-amplified cassettes. There was no significant difference in cell growth and morphology of wild-type and *Mlp1-PrA/Nup84-3FH* yeast cells (Supplementary information, Fig. S2). To gain the high-quality endogenous *sc*NPC, we developed a purification protocol based on previously published methods.^17,43–46^ Briefly, yeast cells were grown at 30 °C in yeast extract peptone dextrose (YPD) medium until reaching the middle log phase (OD600 = 0.8–1.2) and then treated with 10 μg/mL of the α factor for 2 h to synchronize cells in the G1 phase prior to harvest by centrifugation. The pellets of yeast cells were resuspended in lysis buffer (20 mM HEPES-KOH, pH 7.4, 20 mM NaCl, 2 mM MgCl_2_, 50 mM potassium acetate, 0.1% (w/v) Tween-20, 0.5% (w/v) Triton X-100, 10% (v/v) glycerol) at a weight/volume ratio of 1:1, and then frozen in liquid nitrogen followed by cryogenically ground in a mill (ATS Scientific, Freezer/Mill 6875). Frozen cell powder was resuspended in the lysis buffer with 1/100 (w/v) protease inhibitor cocktail (Roche), and gently stirred for 2 h at 4 °C. The lysate was centrifugated at 2500 RCF for 5 min followed by 12,000 RCF for 5 min. The supernatant was collected and incubated with anti-FLAG beads (MERCK, A2220) for 2 h at 4 °C. After extensive wash by lysis buffer, the crude NPCs were eluted by 3× FLAG peptide (Sigma-Aldrich, F4799) in the elution buffer (20 mM HEPES-KOH, pH 7.4, 20 mM NaCl, 2 mM MgCl_2_, 50 mM potassium acetate, 0.1% (w/v) Tween-20, 10% (v/v) glycerol). The eluent was further purified by size exclusive chromatography using a Sephacryl S-500 HR column (GE healthcare, HiPrep^TM^ 16/60 Sephacryl S-500 HR) in the elution buffer. The fractions containing NPCs were pooled together and incubated with the Dynabeads coupled to rabbit IgG antibodies (Sigma-Aldrich, I5006-10 mg) for 1 h at 4 °C. IgG Dynabeads were prepared using the Dynabeads antibody coupling kit (Thermo Fisher, 14311D). After washing with elution buffer, NPCs were released by TEV protease (in-house) cleavage for 2 h at 4 °C in the desired volume of elution buffer. Finally, a magnet was applied to remove the beads and the supernatant containing the *sc*NPC was collected by centrifuging at 20,000× *g* for 10 min.

### Purification of the Nup188, Nup157 and Nup170

DNA fragments of IR subunits were amplified by PCR using *S. cerevisiae* cDNA as template and cloned into corresponding vector for eukaryotic and prokaryotic protein expression. Details of expression constructs are shown in Supplementary information, Table S1. After screening, we focused on purifications of Nup188, Nup157 and Nup170 that displayed the better performances. All subunits were purified with the same following procedure. The plasmids expressing the C-terminally Flag-tagged protein were transformed into *S. cerevisiae* (W303a) cells using the traditional lithium acetate protocol.^47^ Single colonies were picked up and inoculated in 5 mL Synthetic Dextrose (SD) medium containing 100 μg/mL ampicillin and 50 μg/mL kanamycin and grown overnight at 30 °C as the starter culture. In the following morning, the starter culture was added into 1 L of SD medium containing antibiotics for further culture. Cells were grown at 30 °C until the OD600 reached ~2.0–2.5, and then protein production was induced by adding 2% galactose for extra 4–6 h. The yeast cells were harvested by centrifugation at 4800 rpm for 10 min, resuspended in lysis buffer (20 mM HEPES-KOH, pH 7.4, 50 mM potassium acetate, 500 mM NaCl, 2 mM MgCl_2_, 0.1% NP40, 5% (v/v) glycerol, 1/200 (v/v) protease inhibitor cocktail) at a weight/volume ratio of 4:1, frozen in liquid nitrogen and cryogenically ground in the mill. The cell powder was thawed using 2× volumes of lysis buffer and stirred for 1 h. The lysate was cleared by centrifugation at 18,000 rpm for 30 min. The supernatant was incubated with anti-FLAG beads for 2 h at 4 °C. Then the beads were washed extensively by lysis buffer and elution buffer (20 mM HEPES-KOH, pH 7.4, 50 mM potassium acetate, 150 mM NaCl, 2 mM MgCl_2_, 0.01% NP40, 5% (v/v) glycerol) in turn. Proteins were eluted by 200 μg/mL 3× FLAG peptide in the elution buffer. The eluent was applied to a Superose S6 3.2/60 column (GE Healthcare) with running buffer containing 20 mM HEPES-KOH, pH 7.4, 50 mM potassium acetate, 150 mM NaCl and 2 mM MgCl_2_. Peak fractions were collected and concentrated for cryo-EM sample preparation.

### Synchronization of yeast cells

α factor is a well-known pheromone secreted by mating type alpha (*MATα*) cells, and can arrest mating type a (*MATa*) cells in the G1 phase with a clear hallmark of the “Shmoo” shape.^35^ To find the proper concentration of α factor and the time point of cellular arrest, we conducted synchronization experiments of wild-type and *Mlp1-PrA/Nup84-3FH* yeast cells under different conditions. Briefly, overnight cultured yeast cells were diluted to OD600 of ~0.2 followed by continuous shaking for 4–6 h at 30 °C until cells reached the log phase (OD600 = ~0.8). Then the cells were divided into three equal parts, which were added with 5, 10 and 15 μg/mL of α factor, respectively. We monitored the cell cycle arrest by laser scanning confocal microscopy (Olympus, FV1200) every hour (Supplementary information, Fig. S2). Finally, we chose 10 μg/mL of α factor to treat cells for 2 h, because most cells present the “Shmoo” shape under at this condition, and the concentration of the drug is relatively low, which means less toxicity.

### Cryo-EM sample preparation and single-particle data set acquisition

For the NPC, the purified samples with 10% glycerol were checked by negative staining using a Tecnai Spirit at 120 kV (Thermo Fisher Scientific). The most homogenous samples were selected to prepare cryo-EM samples. 200 mesh Au-lacey carbon grids with continuous carbon support film (Electron Microscopy Sciences) were glow discharged in air, and each grid was mounted on forceps in a Mark IV Vitrobot (Thermo Fisher Scientific) at 4 °C and 100% humidity. 4 μL sample drops were floated on the grid for 60 s and then about 3 μL of them were removed by absorbing using pipettor. Then, 4 μL elution buffer without glycerol was added to the grid and the grid was plunge-frozen in liquid ethane after blotting. For Nups (Nup188, Nup157 and Nup170), 4 μL of each purified sample was applied onto a plasma-cleaned holey carbon Au grid (Quantifoil, R1.2/1.3, 300 mesh). Grids were blotted at 100% humidity and 4 °C and then plunge-frozen in liquid ethane using a Mark IV Vitrobot.

Data sets of both NPC complex and Nups (Nup188, Nup157 and Nup170) were collected on a Titan Krios electron microscope operating at 300 kV, equipped with a Gatan K3 Summit direct electron detector and a GIF Quantum energy filter. All data sets were collected using SerialEM^48^ with the same imaging settings. Images were recorded using a pixel size of 0.668 Å at a magnification of 130,000×. The defocus value of each image was set from –1.5 to –2.0 μm during data collection. Each micrograph was dose-fractioned into 32 frames with a total dose of about 50 e^–^/Å^2^.

Finally, 296,820, 16,527, 15,880 and 8451 movies were collected for the NPC, Nup188, Nup157 and Nup170, respectively. The beam-induced motion of the whole micrograph with 32 movie frames was corrected by MotionCor2.^49^ CTF parameters were estimated by Patch CTF estimation in cryoSPARC^50^ for the NPC, Nup157 and Nup170, and were estimated by CTFFIND4^51^ for Nup188. Further details of data collection are given in Supplementary information, Table S2.

### Initial defocus estimation of NPC

To obtain reliable defocus of particles in the micrographs, we used CTFFIND4, Gctf^52^ and patch CTF estimation in cryoSPARC to estimate the CTF parameters. The CTF parameters derived from different software did not show much difference. However, we found that for bad micrographs that contained crystalline ice, contamination or carbon films, the CTF parameters derived from different software were much different. According to the estimations calculated by patch CTF estimation, 187,076 micrographs were selected from 296,820 micrographs with the maximum resolution value below 4 Å, the astigmatism value below 1000 Å and the defocus ranging from 0.4 μm to 4 μm. Considering that the diameter along the equatorial plane of the NPC is huge and each IR monomer of the NPC suffers from large defocus change, we chose patch CTF estimation to determine the CTF model of each micrograph, so that we can use linear interpolation to obtain the local CTF information of each IR monomer and IR dimer based on the CTF model.

### NPC particle picking

We used a convolutional neural network (CNN)-based particle picking software, Topaz,^53^ which can accurately pick the particles including side-view particles and exclude the obvious bad particles. 1731 manually-picked particles from 2000 micrographs were used to train a particle-picking model in Topaz. Because the diameter of the whole NPC particle is very large, in order to speed up the training, we downsample the micrographs by a downsampling factor of 105, which corresponds to the size of the convolution kernel. Finally, 279,900 particles were auto-picked from 187,076 micrographs.

### Generation of the initial model of NPC

The initial model of NPC was generated in cryoSPARC. A subset of 123,769 particles from 279,900 particles were extracted with a box size of 512 pixels (binned 4×, 2.672 Å/pixel). After 2D classification, 11,624 particles were selected to generate an initial model. First, EMD-7321 was low-pass filtered to 30 Å and used as the initial model for Homogeneous Refinement. Then we subtracted the cargo signal from the particles according to the parameters obtained from the refinement. The subtracted particles were subjected to Ab-initio Reconstruction to get 7 models, followed by Heterogeneous Refinement applied with C8 symmetry. Finally, the model with the best resolution (22 Å) was chosen as the initial model.

### Whole NPC complex reconstruction

The image processing was performed in cryoSPARC and RELION3.0/3.1.^54^ 279,900 particles were extracted with a box size of 512 pixels (binned 4×, 2.672 Å/pixel). After 2D classification in cryoSPARC, the obviously bad particles were removed. The remained 263,477 particles were imported into RELION3.0 for 3D classification and refinement. 3D classification was performed with global search, C8 symmetry, *K* = 1 and 50 iterations, and the initial model generated above was low-pass filtered to 30 Å. Then another round of 3D classification with local search, C1 symmetry and *K* = 6 was performed. By this step, we obtained one class showing eight spokes clearly even without applying symmetry. Then the particles from this class were submitted to 3D auto-refine with C8 symmetry to further improve the resolution. The resulted data star file was imported to cryoSPARC to re-extract particles followed by Non-uniform Refinement.^55^ After this step, the signal of center cargo was subtracted from particles by particle subtraction. 2D classification of the subtracted particles indicated that the density of the center cargo was greatly reduced. Finally, we got a whole NPC map at 12.03 Å after Non-uniform Refinement with C8 symmetry imposed.

### IR reconstruction

When we gradually increased the threshold of the whole NPC map, only IR region becomes clearly visible among the three layers, indicating that IR is the most stable and uniform region in the entire *sc*NPC. Thus, we focused on the refinement of IR. Due to the huge diameter, the particle suffers from the large defocus change along the equatorial plane. In order to alleviate this problem, we tried a strategy: refine the CTF and pose parameters of one IR monomer for each NPC particle, and then use the refined parameters to reconstruct the whole IR without alignment. Firstly, local-refinement with an IR monomer mask was performed to further optimize the pose parameters. Then the particles were re-centered to the IR monomer using the refined pose parameters, and 278,938 particles were extracted with a 512-pixel box size (binned 2×, 1.336 Å/pixel) for the IR monomer. After 2D classification, the particles were divided into one good group and one bad group, which were used to generate one good and two bad initial models, respectively. Then, the totally 278,938 particles were subjected to Heterogeneous Refinement with the three initial models generated above. The class with best quality was further subjected to Non-uniform Refinement, which resulted in an 8.22 Å map with 101,757 particles. A round of CTF refinement and Non-uniform Refinement was performed. Finally, the resolution was improved to 7.41 Å. Then, 101,757 particles were re-centered to the center of the whole NPC and extracted with a 512-pixel box size (binned 4×, 2.672 Å/pixel) for the whole NPC. The refined CTF parameters and pose information obtained from the 7.41 Å map of the IR monomer were applied back to the corresponding whole NPC particles for reconstruction without alignment and with an IR mask and C8 symmetry imposed, which resulted in a 13 Å IR map. After post-processing with an IR mask and B-factor of –800, we obtained an entire IR map at 9.10 Å.

### IR monomer/protomer and IR dimer reconstruction

In order to further improve the resolution of the IR monomer, we locally refined each IR monomer of whole NPC, then used the corresponding pose parameters to extract each IR monomer particle with a 350-pixel box size (binned 2×, 1.336 Å/pixel). The extracted 2,238,689 particles were subjected to 2D classification to remove obvious bad particles that were used to generate four bad initial models by Ab-initio Reconstruction. The remained 1,954,403 particles were used to reconstruct a good initial model by Reconstruction only. A round of Non-uniform Refinement and CTF refinement was performed. Then Heterogeneous Refinement with five initial models generated above was performed to remove bad particles. 795,543 particles allocated to the best class were subjected to Non-uniform Refinement. The resolution was improved to 3.98 Å without applying symmetry and 3.84 Å with C2 symmetry imposed. Using the 3.84 Å map together with four bad initial models, the second round of Heterogeneous Refinement was performed to screen out more bad particles from the initial 1,954,403 particles. 679,756 particles of the best class were selected for 2D classification and Non-uniform Refinement. We obtained a 3.73 Å map with 633,134 particles. In order to further improve the resolution to aid model building, we performed symmetry expansion of each IR monomer particle. Each monomer was replicated and rotated two times around its C2 (pseudo) symmetry axis, followed by signal subtraction to retain one copy of each protomer, and subsequent local refinement. Although the final resolution of protomer is 3.71 Å with 2-fold particles, only slightly higher than that of IR monomer, the map quality of CNT is much better than that in the IR monomer.

In order to improve the quality of the density at the connection region between adjacent IR monomers, we used the similar methods as above to obtain an IR dimer map at 7.69 Å.

All reported resolutions were estimated based on the gold-standard Fourier Shell Correlation (FSC) 0.143 criterion.^54^ All local resolution maps were determined using cryoSPARC.

### Image processing of Nup157, Nup170 and Nup188

For Nup157, a small dataset of 88,788 particles were picked from 200 micrographs using blob picker with particle diameters ranging from 70 Å to 200 Å and processed by reference-free 2D classification using cryoSPARC. 8421 good particles with clear 2D averages were selected as training dataset for Topaz, by which a total of 2,652,917 particles were automatically picked from all micrographs. After 3 rounds of 2D classification, 1,993,084 particles were selected and subjected to 2 rounds of Ab-initio Reconstruction with 6 classes in cryoSPARC. Among these classes in the second round, class I with the clearest and the most complete density was selected for Homogeneous Refinement in cryoSPARC. Another round of 2D classification was executed for removing the incomplete particles lacking C-terminal region, and 100,523 particles were selected and imported into RELION3.0. After 3D auto-refinement and post-processing, the final resolution of the full-length Nup157 density map is 5.9 Å. To analyze the particle heterogeneity, the 100,523 particles were imported into EMAN2.91,^56^ and subjected to 2D classification. One class was chosen as the 2D reference. All the selected particles were aligned to the single 2D reference using e2a2d_align.py command, and further analyzed by e2motion.py command. The N-terminal regions were used for realignment of the particles and the C-terminal regions were used for classification. Finally, 32 images of 2D class average were generated and used to make the animated movie (Supplementary information, Video S3).

For Nup170, a small dataset of 59,240 particles were picked from 200 micrographs using blob picker with particle diameters ranging from 70 to 200 Å and processed by reference-free 2D classification using cryoSPARC. 6700 good particles with clear 2D averages were selected as training dataset for Topaz, by which a total of 1,733,609 particles were automatically picked from all micrographs. After 2 rounds of 2D classification, 944,829 particles were selected and subjected to Ab-initio Reconstruction with 5 classes in cryoSPARC. We failed to get full-length Nup170 map after trying many methods, and one class (130,369 particles) with the clearest density was selected for Non-uniform Refinement in cryoSPARC. Then particles were imported into RELION3.0, and CTF refinement, 3D auto-refinement and post-processing were applied. The final resolution of the Nup170 density map is 4.09 Å.

For Nup188, 16,527 micrographs were selected with the maximum resolution value below 4 Å and the astigmatism below 1000 Å. The following image processing steps were carried out in cryoSPARC and RELION 3.1 as shown in Supplementary information, Fig. S12. 5,610,043 particles were auto-picked using blob picker with particle diameters ranging from 100 Å to 170 Å and subjected to 2D classification. 1,144,198 particles with good class-averages were selected and subjected to Ab-initio Reconstruction for 10 classes without reference. Two classes (672,071 particles) with good structure features were selected and another Ab-initio Reconstruction was performed for the other 8 classes without reference. Five classes (607,216 particles) with good structure features were selected and subjected to Non-uniform Refinement with one good initial structure reconstructed above as reference, leading to a 3D map at an overall resolution of 2.81 Å. All these particles were imported into RELION 3.1, and auto-refined using the 2.81 Å map low-pass filtered to 60 Å as reference to reconstruct a map at an overall resolution of 3.02 Å after post-processing. A soft mask was applied for further auto-refinement, and a 3D reconstruction at an overall resolution of 2.86 Å was obtained after post-processing. In the map, the density of the C-terminal part (amino acids 1216–1653) was much weaker than the N-terminal part.

To analyze the particle heterogeneity, all the 607,216 particles were imported into EMAN2.91,^56^ and subjected to 2D classification. All top view classes (148,412 particles) with clear secondary structure features were selected and one of them was chosen as the 2D reference. All the selected particles were aligned to the single 2D reference using e2a2d_align.py command, and further analyzed by e2motion.py command. The N-terminal regions were used for realignment of the particles and the C-terminal regions were used for classification. Finally, 48 images of 2D class average were generated and 46 images of them were selected to make the animated movie (Supplementary information, Video S1).

To describe the continuous structural heterogeneity of the C-terminal region in Nup188, two soft masks were made for N-terminal and C-terminal regions according to the 3.02 Å map low-pass filtered to 15 Å, respectively. All the 607,216 particles were subjected to multi-body refinement in RELION 3.1, and 3.02 Å and 3.41 Å reconstructions for the N-terminal and C-terminal regions were yielded, respectively (Supplementary information, Fig. S12). The movie of C-terminal region motions was generated (Supplementary information, Video S2).

All reported resolutions were estimated based on the gold-standard FSC 0.143 criterion. All local resolutions were calculated using RELION3.0.

### Model building and refinement

Atomic coordinates of Nup188 were generated utilizing map-to-model software in PHENIX^57^ with homologous crystal structures as the templates, including the N-terminal region (PDB: 5CWU and 4KF7) and C-terminal region (PDB: 4KF8), and manually refined in COOT^58^ based on density of Nup188 determined in this study. The crystal structure of the N-terminal region (PDB: 4MHC) and the predicted C-terminal model of Nup157 were combined and fitted into both the 5.9 Å map of the full-length Nup157 and the 3.73 Å map of the IR monomer to gain a composite model of Nup157. The N-terminal model generated by SWISS-MODEL^59^ and the C-terminal crystal structure (PDB: 3I5P) of Nup170 were manually adjusted based on the maps of Nup170, IR monomer and IR dimer to gain a composite model of Nup170. Model of Nic96 was from crystal structure (PDB: 2RFO) lacking the N-terminal disordered region. The atomic models of Nup192 and CNT complex were generated through homology modeling using SWISS-MODEL and I-TASSER^60^ and adjusted based on the EM density map of IR monomer. All atomic coordinates for the individual subunits of IR were firstly fitted into the EM density map of the IR monomer based on the IR model from^18^ (PDBDEV_00000051) using UCSF Chimera^61^ and Chimera X^62^ and then manually adjusted according to the EM density maps of IR monomer and IR dimer in COOT. Real space refinement and final validation of IR monomer model were performed through real space refinement and validation program from PHENIX. The final atomic model of Nup188 was cross-validated according to previously described procedures.^63^ Briefly, atoms in the model were randomly shifted by up to 0.5 Å, and then refined against one of the two independent half maps generated during the final 3D reconstruction. Then, the refined model was tested against the other map. FSC curves of the refined model versus the overall map (sum, blue), of the model refined against the first half map versus that same map (work, red), and of the model refined against the first half map versus the second map (free, green) were shown in Supplementary information, Fig. S12. Structural visualizations and Figures were performed by UCSF Chimera, Chimera X and PyMOL (www.pymol.org).

## Supplementary information

Supplementary information, Fig. S1

Supplementary information, Fig. S2

Supplementary information, Fig. S3

Supplementary information, Fig. S4

Supplementary information, Fig. S5

Supplementary information, Fig. S6

Supplementary information, Fig. S7

Supplementary information, Fig. S8

Supplementary information, Fig. S9

Supplementary information, Fig. S10

Supplementary information, Fig. S11

Supplementary information, Fig. S12

Supplementary information, Fig. S13

Supplementary information, Fig. S14

Supplementary information, Fig. S15

Supplementary information, Fig. S16

Supplementary information, Fig. S17

Supplementary information, Table S1

Supplementary information, Table S2

Supplementary information, Video legend

Supplementary Video S1

Supplementary Video S2

Supplementary Video S3

Supplementary Video S4

## Data Availability

The EM density maps of the intact NPC (EMDB: EMD-32664), the intact IR (EMDB: EMD-32663), the IR dimer (EMDB: EMD-32662), the IR monomer (EMDB: EMD-32658), the IR protomer (EMDB: EMD-32653), Nup157 (EMDB: EMD-32668), the N-terminal region of Nup170 (EMDB: EMD-32677) and Nup188 (EMD-32643 (the full-length Nup188), EMD-32644 (the N-terminal region of Nup188), EMD-32645 (the C-terminal region of Nup188)) have been deposited in the Electron Microscopy Data Bank (www.ebi.ac.uk/pdbe/emdb/). Atomic coordinates of the IR monomer (PDB: 7WOT), the IR protomer (PDB: 7WOO), Nup188 (PDB: 7WO9), Nup157 (PDB: 7WP4) and Nup170 (PDB: 7WP7) have been deposited in the Protein Data Bank (www.rcsb.org). All other data and materials are available from the corresponding authors upon reasonable request.
