# Supplementary material for: Near-atomic structure of the inner ring of the Saccharomyces cerevisiae nuclear pore complex
Source: Cell Res. 2022 Mar 18;32(5):437–50. doi: 10.1038/s41422-022-00632-y (PMC9061825; doi:10.1038/s41422-022-00632-y)
Supplement: Supplementary file 1 — Supplementary information, Fig. S1 [file 41422_2022_632_MOESM1_ESM.pdf]

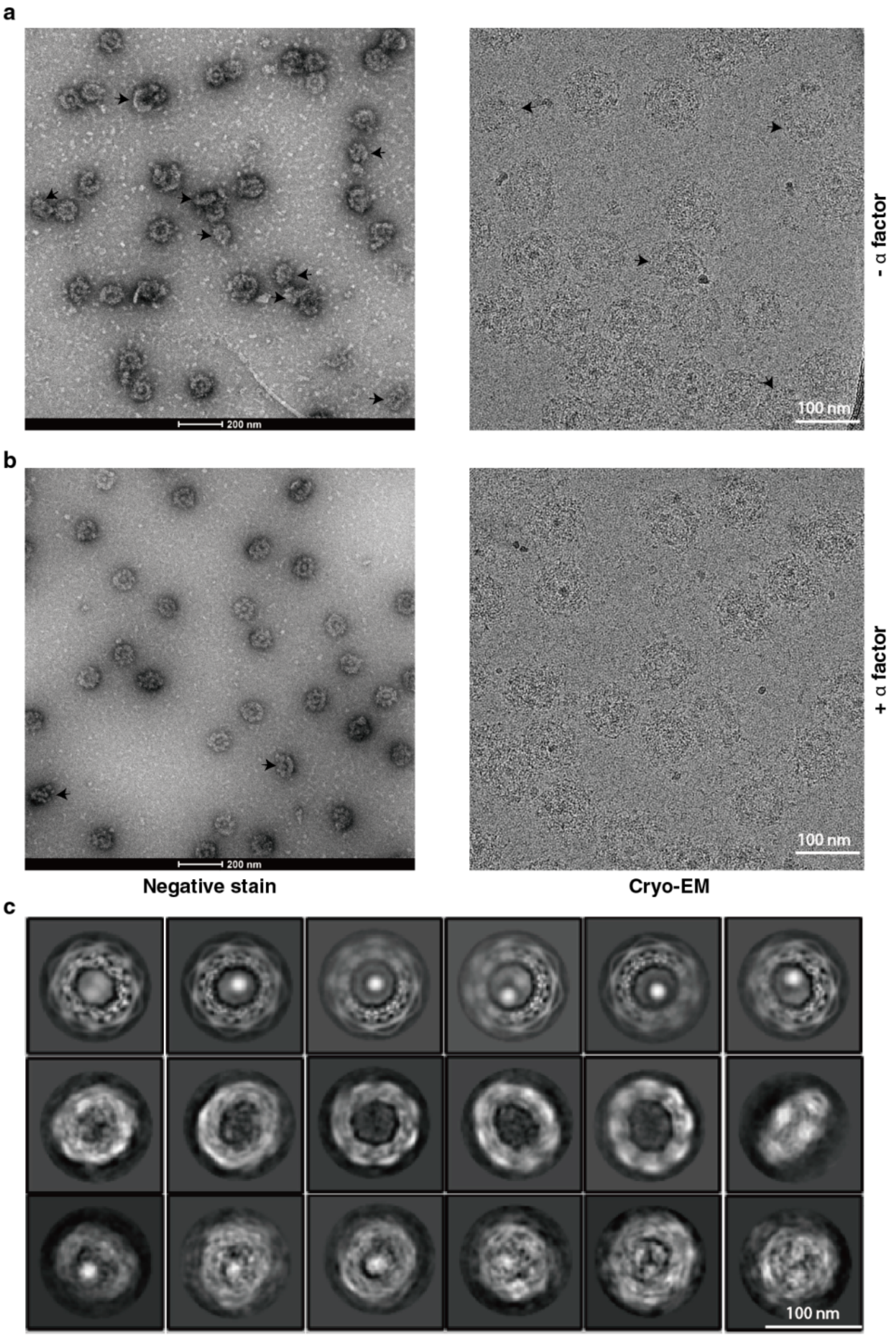

Supplementary information, Fig. S1. Structural variability of *sc*NPC.
(**a**, **b**) Representative negative staining EM and cryo-EM micrographs of *sc*NPC treated with or without alpha-mating factor. Heterogeneous particles are indicated by black arrows. (**c**) Typical reference-free 2D class averages of *sc*NPC with the different conformations and sizes.
