# Supplementary material for: Near-atomic structure of the inner ring of the Saccharomyces cerevisiae nuclear pore complex
Source: Cell Res. 2022 Mar 18;32(5):437–50. doi: 10.1038/s41422-022-00632-y (PMC9061825; doi:10.1038/s41422-022-00632-y)
Supplement: Supplementary file 2 — Supplementary information, Fig. S2 [file 41422_2022_632_MOESM2_ESM.pdf]

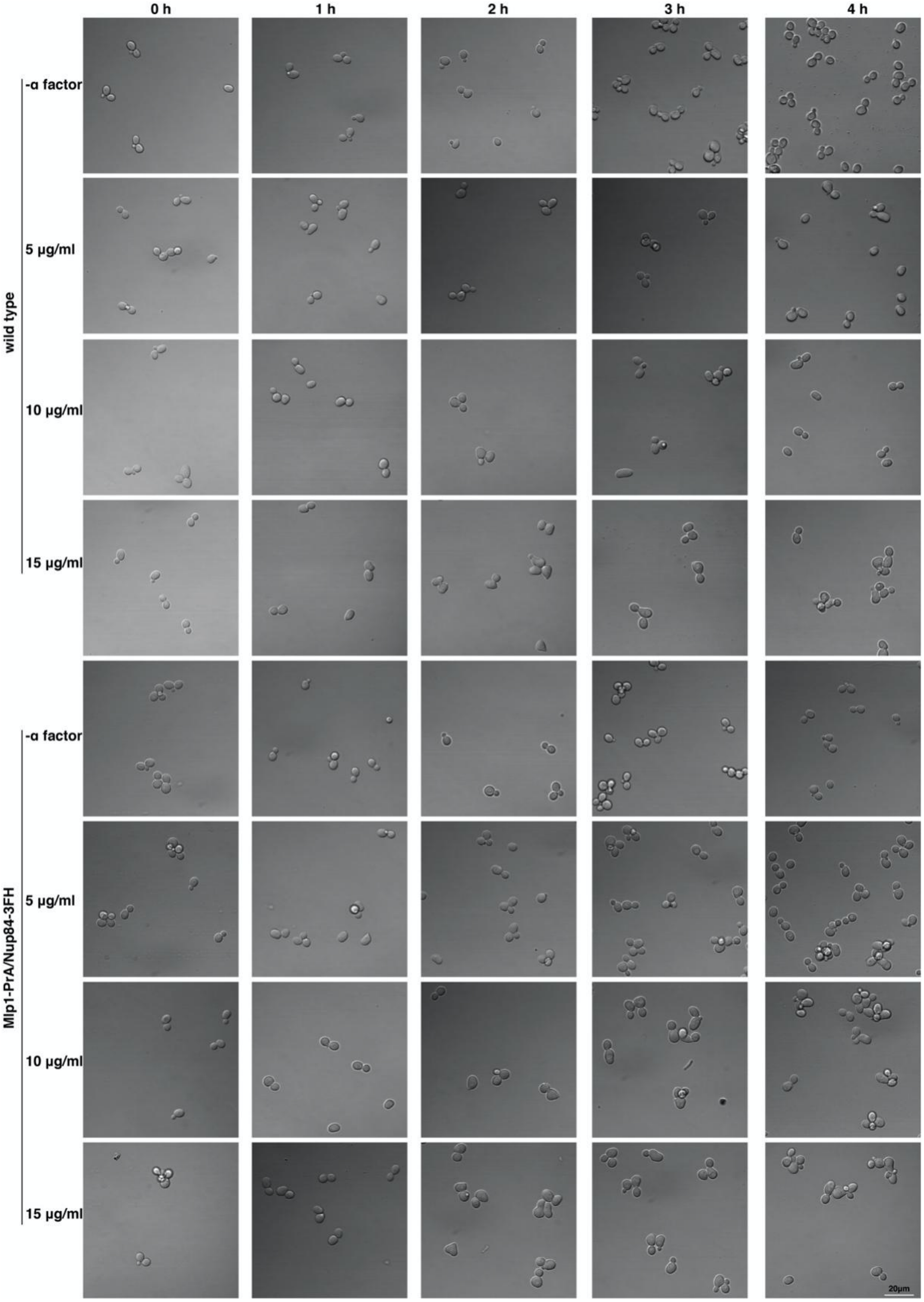

Supplementary information, Fig. S2. Effect of alpha-mating factor on yeast cells.
Alpha-mating factor induced morphologic changes of wild-type and *Mlp1-PrA/Nup84-3FH* yeast cells. Different concentrations and treatment times were experimented and observed on laser scanning confocal microscope (Olympus FV1200). Shmoo shapes suggest cells are arrested in G1 phase.
