# Supplementary material for: Near-atomic structure of the inner ring of the Saccharomyces cerevisiae nuclear pore complex
Source: Cell Res. 2022 Mar 18;32(5):437–50. doi: 10.1038/s41422-022-00632-y (PMC9061825; doi:10.1038/s41422-022-00632-y)
Supplement: Supplementary file 3 — Supplementary information, Fig. S3 [file 41422_2022_632_MOESM3_ESM.pdf]

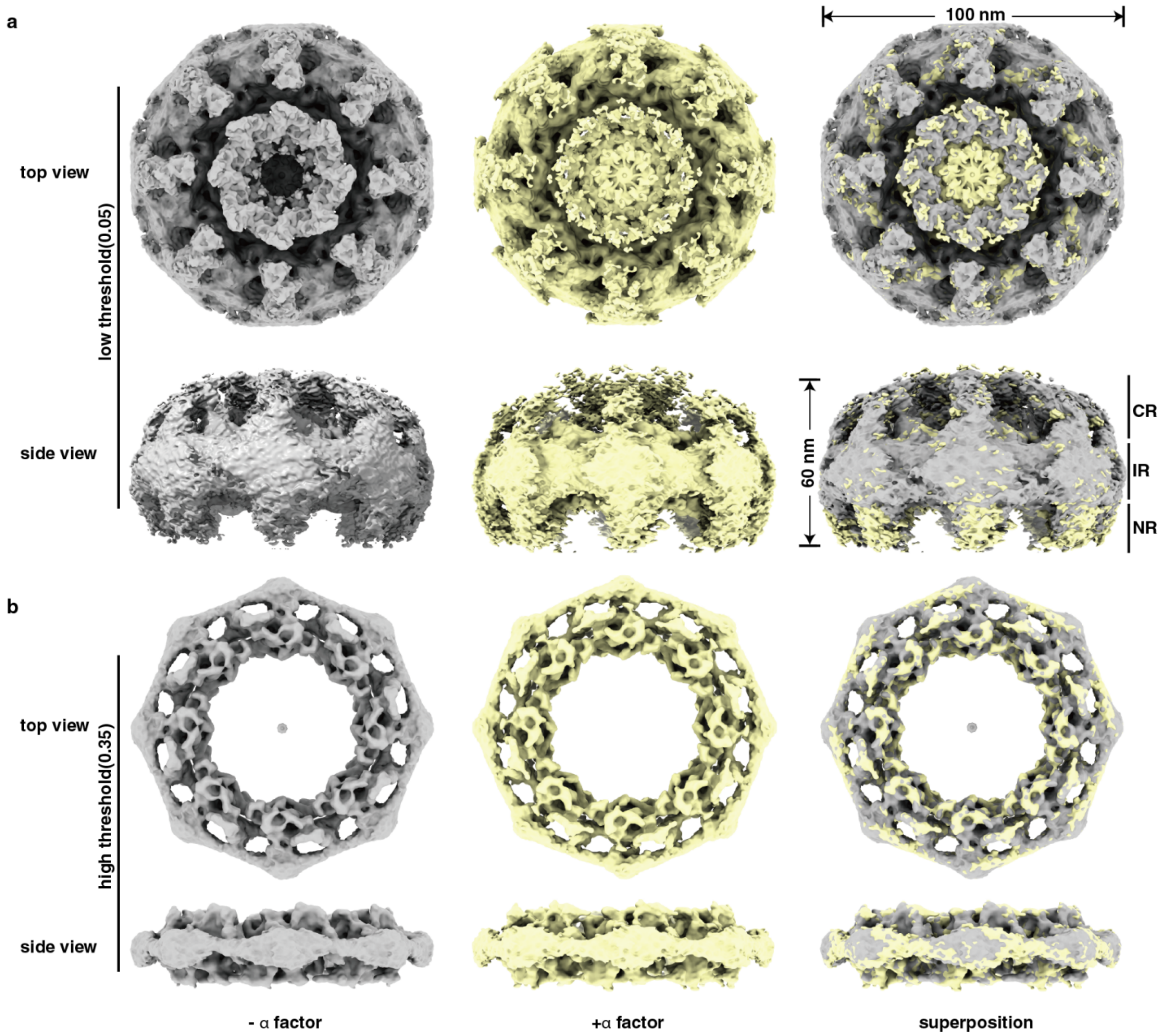

Supplementary information, Fig. S3. Structures of NPC extracted from yeast cells treated with or without alpha-mating factor.
(**a**, **b**) EM density maps are shown in low (a) and high (b) threshold, and superpositions are shown in the right panels.
