# Supplementary material for: Near-atomic structure of the inner ring of the Saccharomyces cerevisiae nuclear pore complex
Source: Cell Res. 2022 Mar 18;32(5):437–50. doi: 10.1038/s41422-022-00632-y (PMC9061825; doi:10.1038/s41422-022-00632-y)
Supplement: Supplementary file 5 — Supplementary information, Fig. S5 [file 41422_2022_632_MOESM5_ESM.pdf]

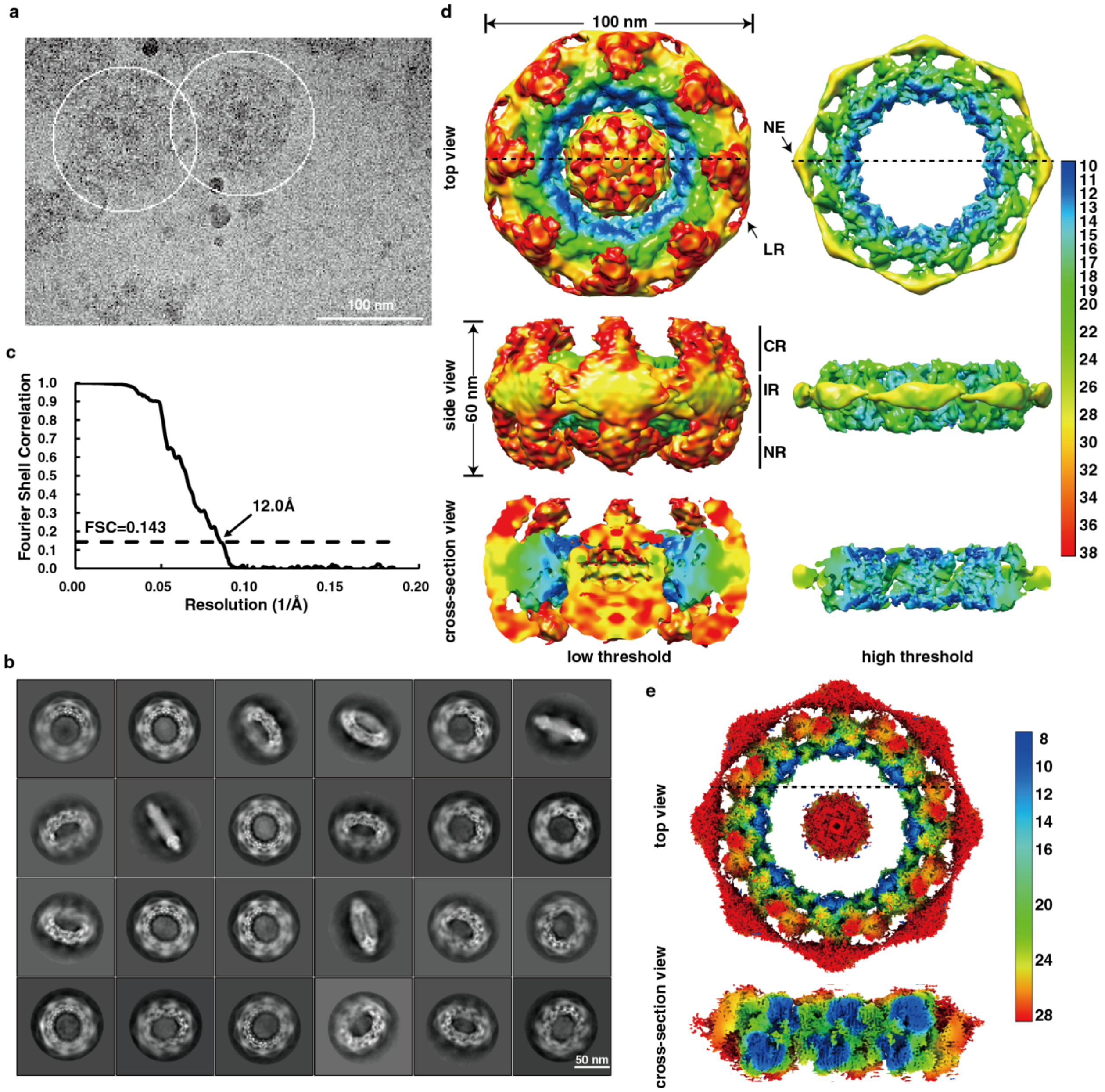

Supplementary information, Fig. S5. Cryo-EM data analysis of entire NPC and entire IR. (**a**) A representative raw cryo-EM image of NPC. (**b**) Typical good reference-free 2D class averages of NPC. (**c**) Gold standard FSC curve for the cryo-EM map of entire NPC. (**d**) Local resolutions of cryo-EM map for entire NPC with different threshold. (**e**) Local resolutions of cryo-EM map for intact IR at different views. Dotted line indicates the cross section corresponding to the side view.
