# Supplementary material for: Near-atomic structure of the inner ring of the Saccharomyces cerevisiae nuclear pore complex
Source: Cell Res. 2022 Mar 18;32(5):437–50. doi: 10.1038/s41422-022-00632-y (PMC9061825; doi:10.1038/s41422-022-00632-y)
Supplement: Supplementary file 6 — Supplementary information, Fig. S6 [file 41422_2022_632_MOESM6_ESM.pdf]

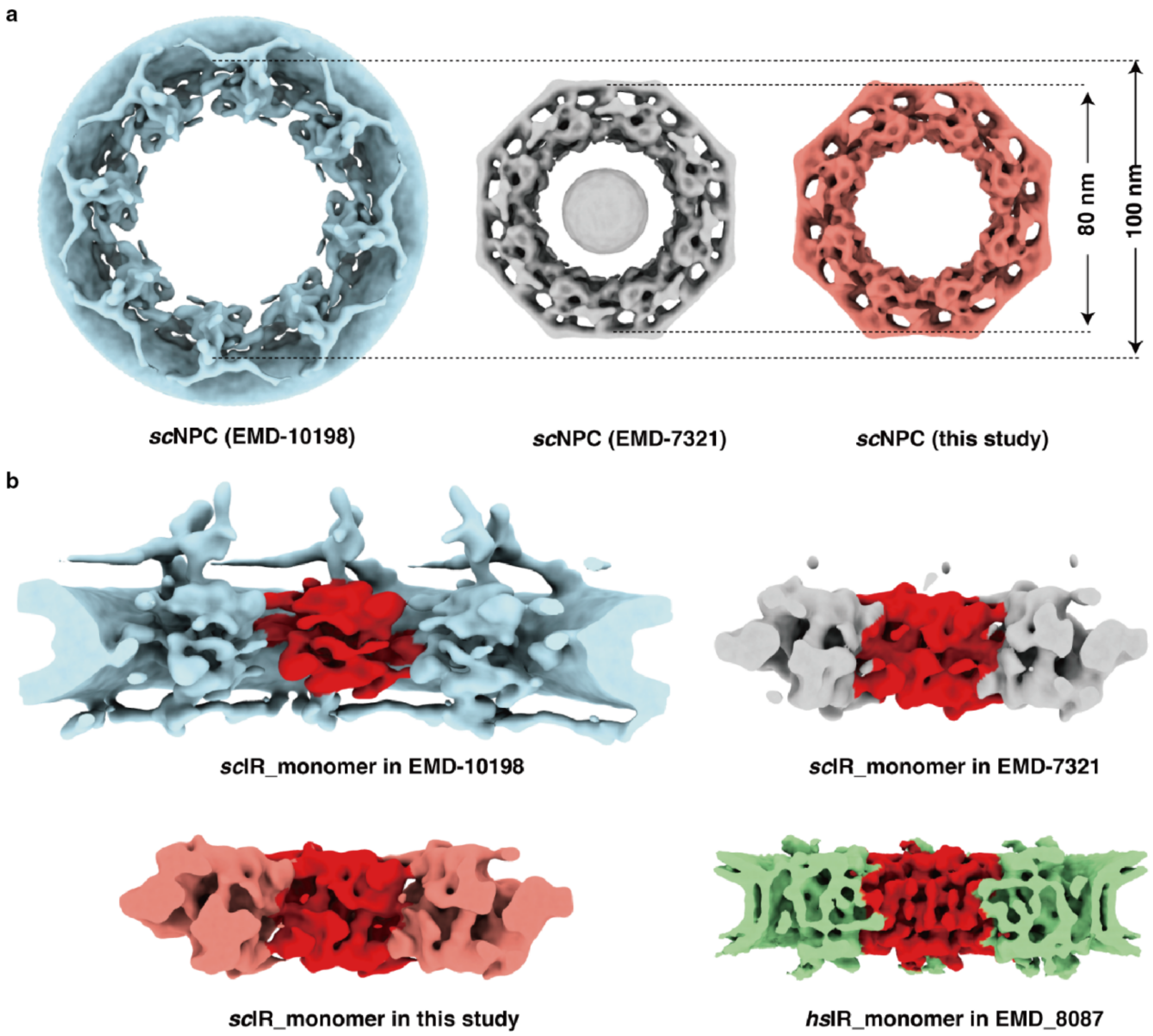

Supplementary information, Fig. S6. The changeable intact IR and relatively stable IR monomer.

(**a**) Comparison of *S. cerevisiae* IR from *in situ* architecture (EMD-10198) (34), detergent-extracted (EMD-7321) (27) and this study indicates ~ 20 nm diameter difference. (**b**) Comparison of IR monomer from different cell states and species including *S. cerevisiae* IR monomer at the active transport state (EMD-10198) and the static state (EMD-7321 and this study), and *Homo sapiens* IR monomer (EMD_8087).
