# Supplementary material for: Near-atomic structure of the inner ring of the Saccharomyces cerevisiae nuclear pore complex
Source: Cell Res. 2022 Mar 18;32(5):437–50. doi: 10.1038/s41422-022-00632-y (PMC9061825; doi:10.1038/s41422-022-00632-y)
Supplement: Supplementary file 7 — Supplementary information, Fig. S7 [file 41422_2022_632_MOESM7_ESM.pdf]

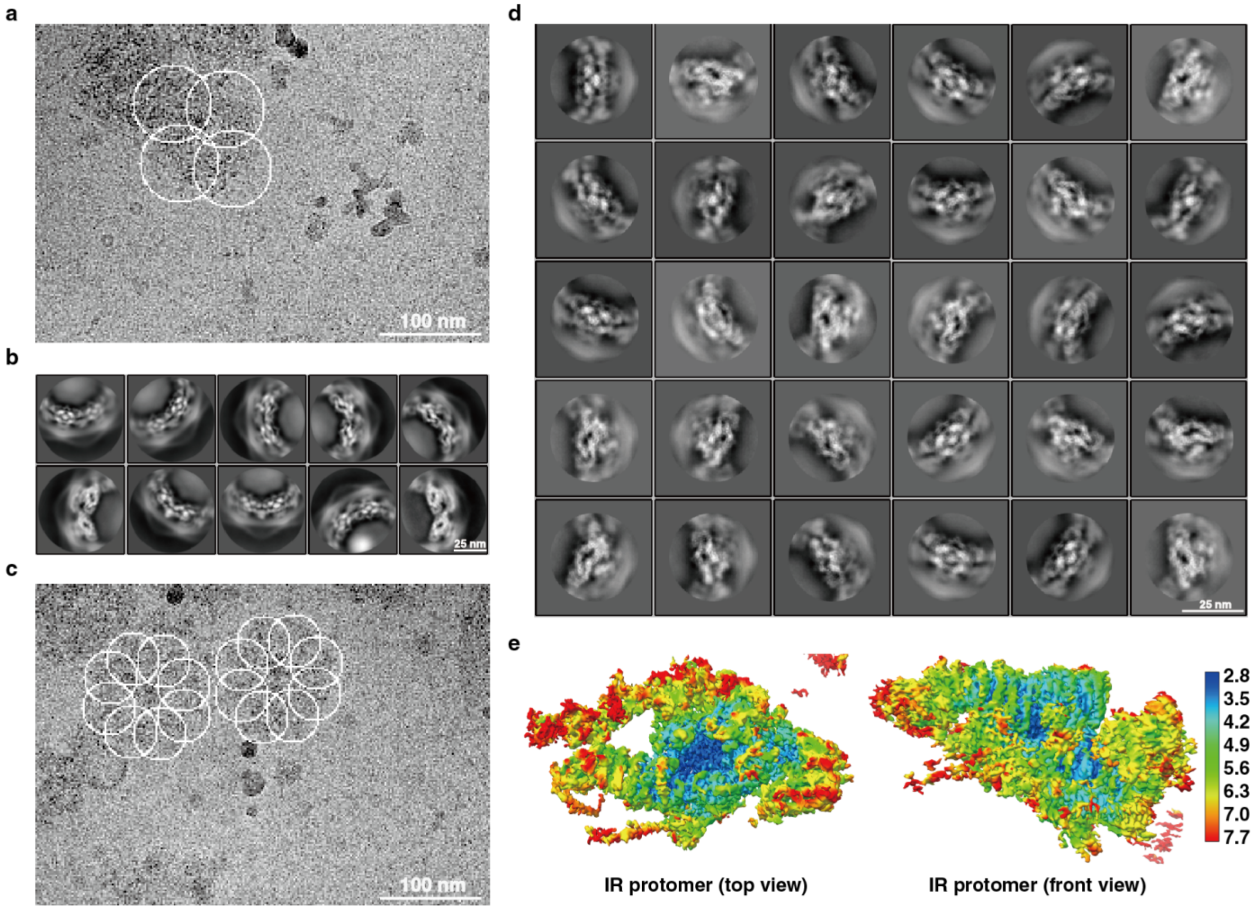

Supplementary information, Fig. S7. Cryo-EM data analysis of IR dimer, monomer and protomer.

(**a**) A representative raw cryo-EM image of NPC labeled with circles for IR dimer particle extraction. (**b**) Typical good reference-free 2D class averages of IR dimer. (**c**) A representative raw cryo-EM image of NPC labeled with circles for IR monomer particle extraction. (**d**) Typical good reference-free 2D class averages of IR monomer. (**e**) Local resolutions of cryo-EM map for IR protomer at different views.
