# Supplementary material for: Near-atomic structure of the inner ring of the Saccharomyces cerevisiae nuclear pore complex
Source: Cell Res. 2022 Mar 18;32(5):437–50. doi: 10.1038/s41422-022-00632-y (PMC9061825; doi:10.1038/s41422-022-00632-y)
Supplement: Supplementary file 8 — Supplementary information, Fig. S8 [file 41422_2022_632_MOESM8_ESM.pdf]

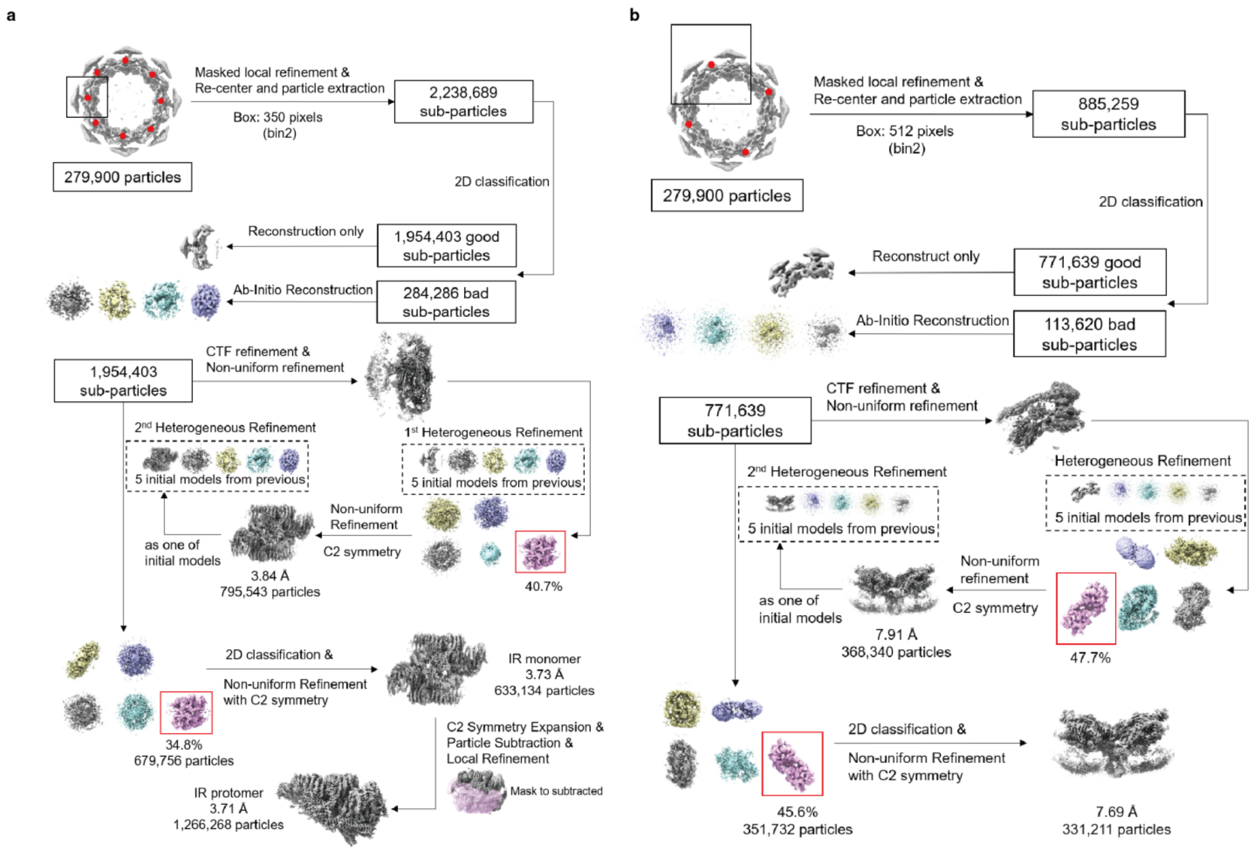

Supplementary information, Fig. S8. Flowcharts for cryo-EM data processing of IR dimer (a), monomer and protomer (b).
See "Materials and Methods" for details.
