# Supplementary material for: Near-atomic structure of the inner ring of the Saccharomyces cerevisiae nuclear pore complex
Source: Cell Res. 2022 Mar 18;32(5):437–50. doi: 10.1038/s41422-022-00632-y (PMC9061825; doi:10.1038/s41422-022-00632-y)
Supplement: Supplementary file 9 — Supplementary information, Fig. S9 [file 41422_2022_632_MOESM9_ESM.pdf]

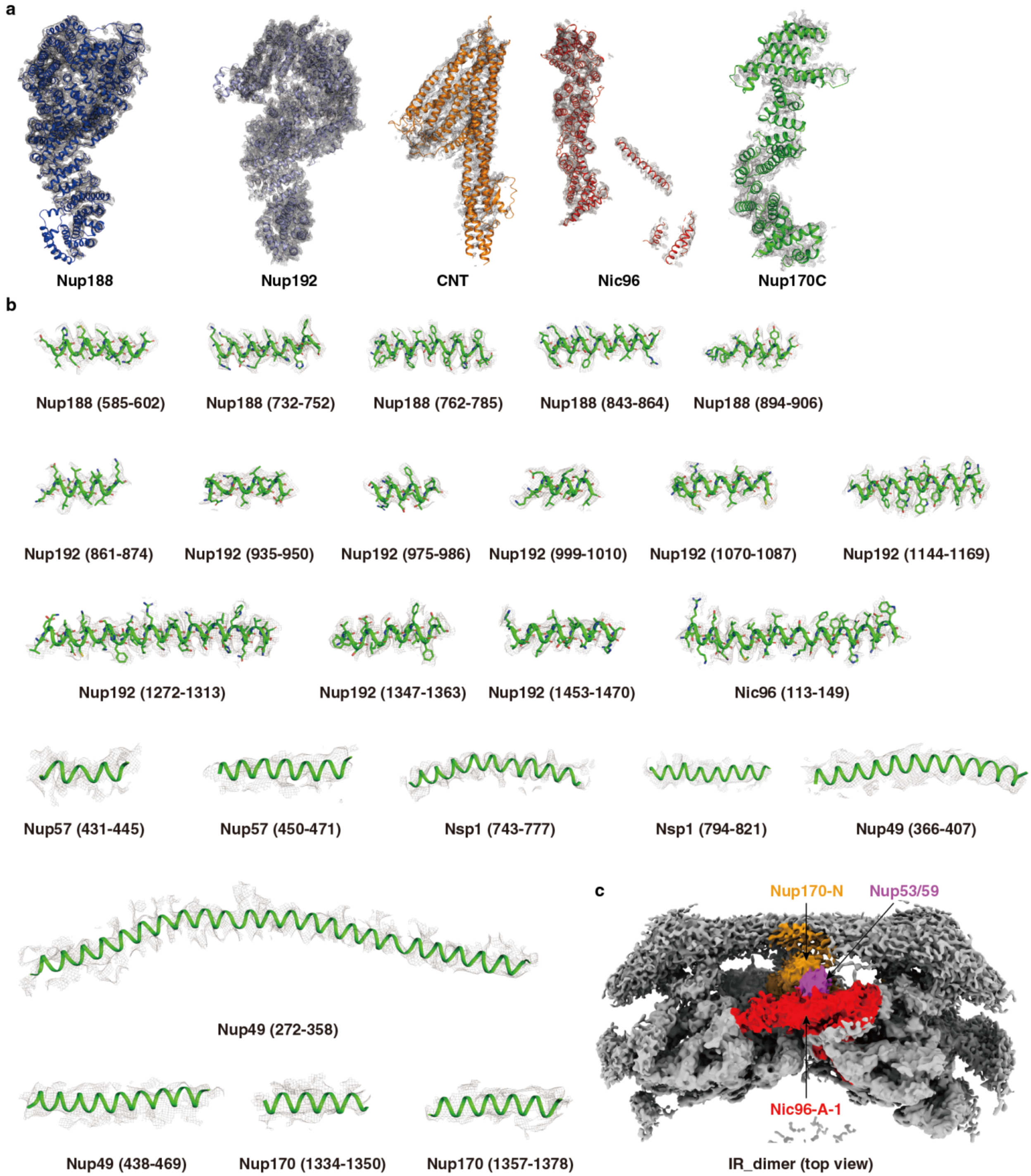


**Supplementary information, Fig. S9. Representative density maps of IR subunits.**
(**a**) The overall EM density map of IR subunits. (**b**) Representative EM density maps for a series of discrete α-helices from IR subunits. (**c**) Location of Nup53/Nup59 density based on our map and previously published biochemical results.
