# Supplementary material for: Near-atomic structure of the inner ring of the Saccharomyces cerevisiae nuclear pore complex
Source: Cell Res. 2022 Mar 18;32(5):437–50. doi: 10.1038/s41422-022-00632-y (PMC9061825; doi:10.1038/s41422-022-00632-y)
Supplement: Supplementary file 10 — Supplementary information, Fig. S10 [file 41422_2022_632_MOESM10_ESM.pdf]

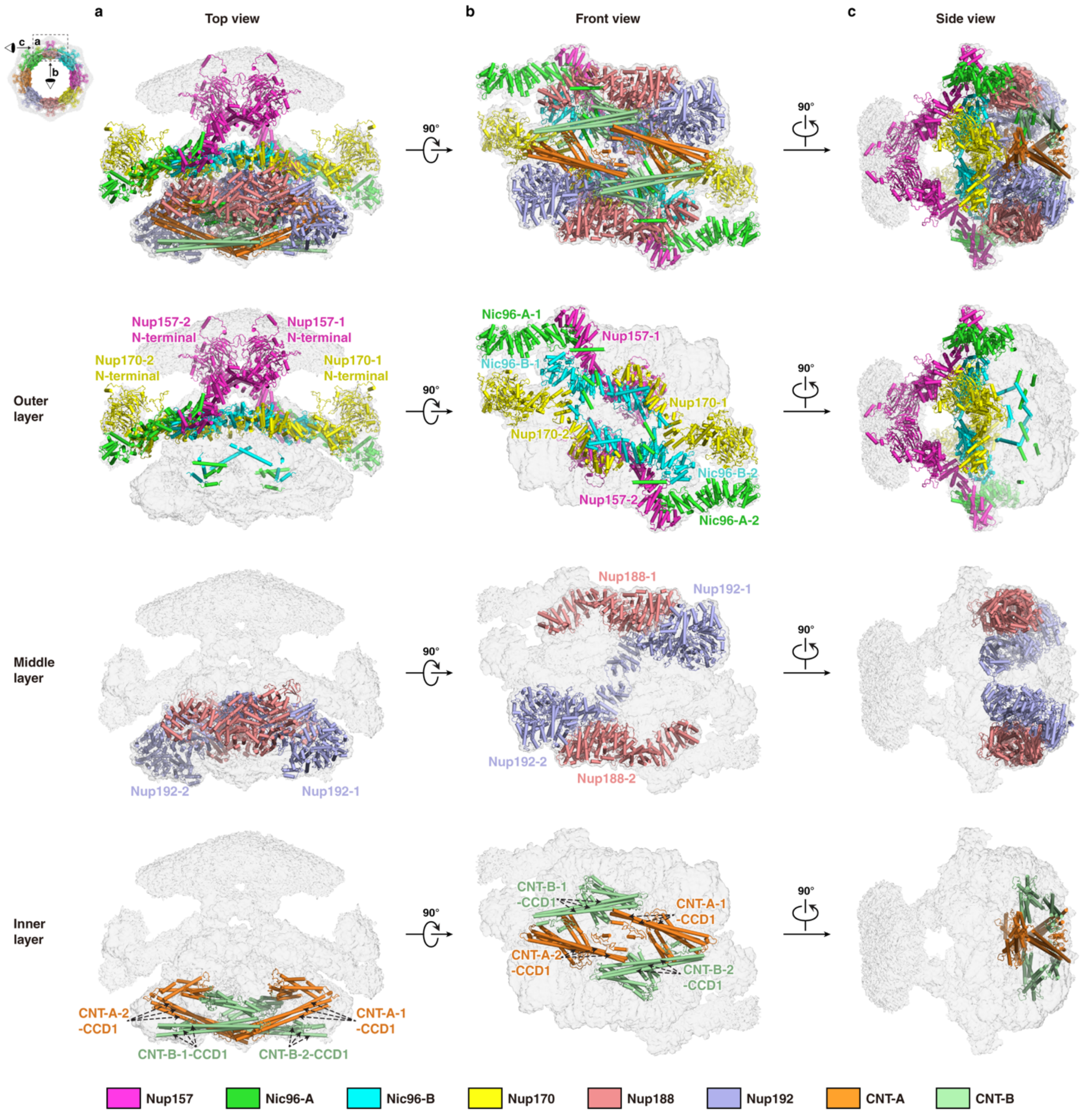

Supplementary information, Fig. S10. Overall organization of the IR monomer.
(**a-c**) Overall structures of the IR monomer and each layer are shown in three perpendicular views. The IR monomer is divided into three layers: outer layer, middle layer and inner layer. The subunits of IR are color coded and shown in cartoon representation. IR monomer includes 24 proteins from 8 different Nups, Nup157, Nup170, Nic96, Nup88, Nup192, Nsp1, Nup49 and Nup57. The last three proteins (Nsp1, Nup49 and Nup57) form CNT complex.
