# Supplementary material for: Near-atomic structure of the inner ring of the Saccharomyces cerevisiae nuclear pore complex
Source: Cell Res. 2022 Mar 18;32(5):437–50. doi: 10.1038/s41422-022-00632-y (PMC9061825; doi:10.1038/s41422-022-00632-y)
Supplement: Supplementary file 11 — Supplementary information, Fig. S11 [file 41422_2022_632_MOESM11_ESM.pdf]

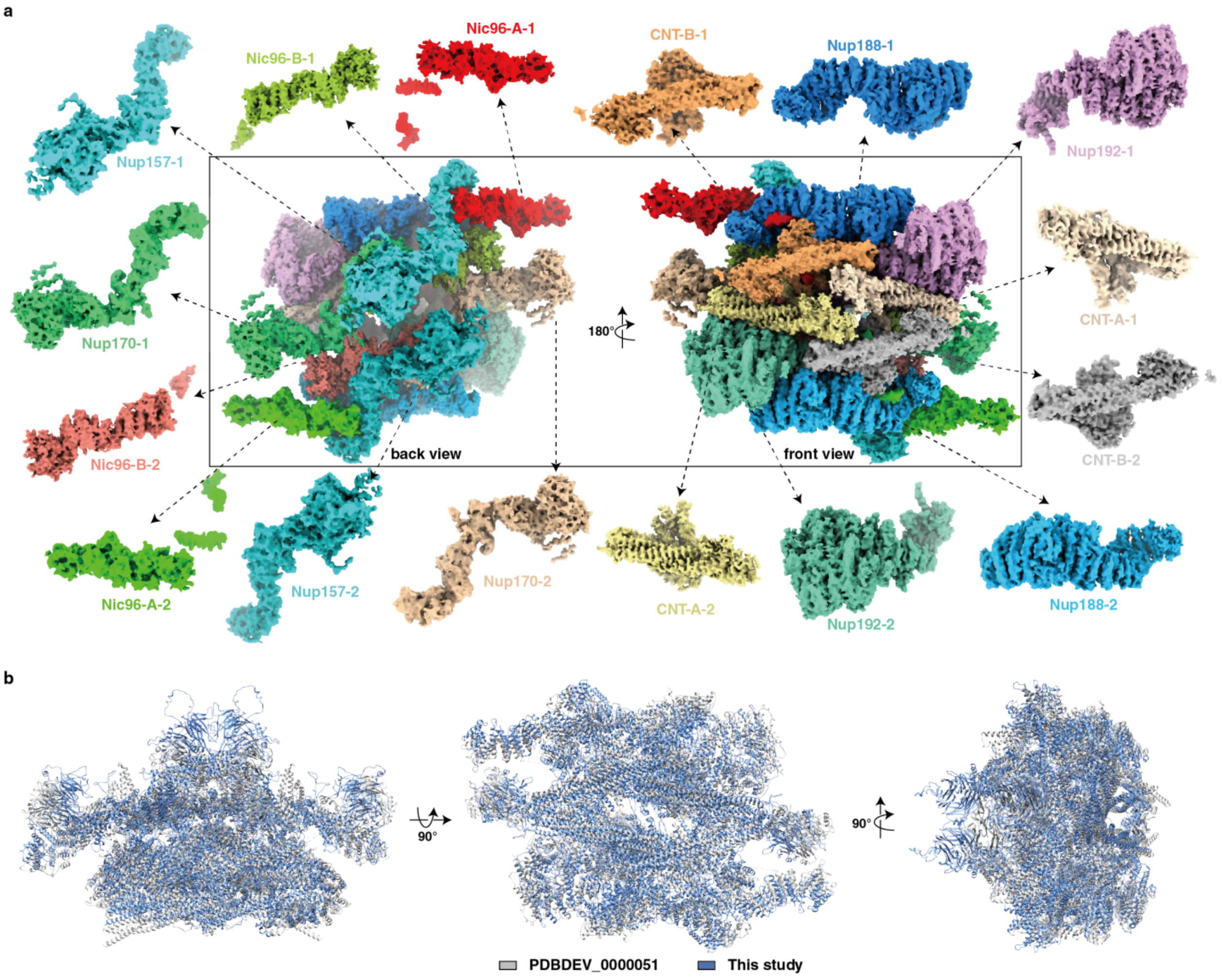

Supplementary information, Fig. S11. Architecture of IR monomer.
(**a**) The density map of IR monomer is shown in two views and 24 proteins from IR monomer are color-coded. CNT complex, which is composed of three proteins, Nup57, Nup49 and NSP1, is painted as the same color. (**b**) Structural comparison of IR monomer in this study and reported previously.
