# Supplementary material for: Near-atomic structure of the inner ring of the Saccharomyces cerevisiae nuclear pore complex
Source: Cell Res. 2022 Mar 18;32(5):437–50. doi: 10.1038/s41422-022-00632-y (PMC9061825; doi:10.1038/s41422-022-00632-y)
Supplement: Supplementary file 12 — Supplementary information, Fig. S12 [file 41422_2022_632_MOESM12_ESM.pdf]

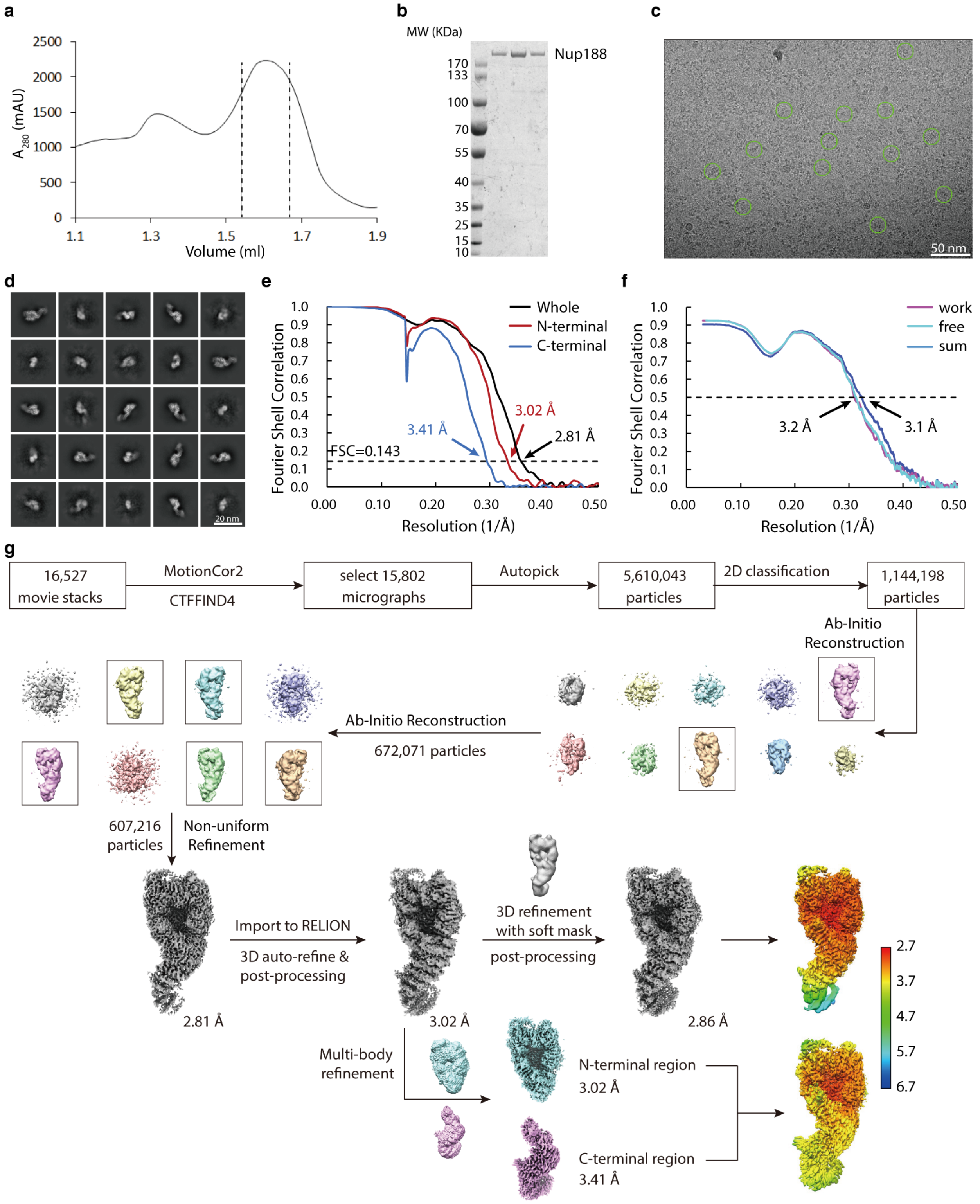

Supplementary information, Fig. S12. Cryo-EM data analysis of Nup188.
(**a**, **b**) Purification of Nup188. SEC profile of Nup188 (a) and the SDS-PAGE gel of the fractions corresponding to the region between dashed lines on SEC curve (b). (**c**) A representative raw cryo-EM image for Nup188 with typical particles marked by green circles. (**d**) Typical good reference-

free 2D class averages of Nup188. (**e**) Gold standard FSC curves for the cryo-EM maps of whole Nup188, its N-terminal part and its C-terminal part. (**f**) FSC curves of the cross-validation of the Nup188 model. See “Materials and Methods” for details. The small difference between the red and green curves indicates that the refinement of the atomic coordinates was not affected by overfitting. (**g**) The flowchart for EM data processing and the local-resolution maps. Details can be found in “Materials and Methods”.
