# Supplementary material for: Near-atomic structure of the inner ring of the Saccharomyces cerevisiae nuclear pore complex
Source: Cell Res. 2022 Mar 18;32(5):437–50. doi: 10.1038/s41422-022-00632-y (PMC9061825; doi:10.1038/s41422-022-00632-y)
Supplement: Supplementary file 13 — Supplementary information, Fig. S13 [file 41422_2022_632_MOESM13_ESM.pdf]

a

Nup188

α1 α2 α3 α4 α5

Nup188 1 MATPSFGNSSPQLTFTHVANFMNDAAADVSAVDAKQLAQIRQFLKANKTNLIESLNTIRQNVTSSGDHNKLRSTIANLLQINVDNDPFFA

α6 α7 α8 α9

Nup188 91 QSEDLSHAVEFFMSERSSRLHIVYSLLVNPDIDLETYSFIDNDRFNVVGKLISIISSVIQNYDIITASSLAHDYNNDQDMFTIVSLVQLK

α10 α11 α12

Nup188 181 KFSDLKFILQILQILNLMILNTKVPVDIVNQWFLQYQNQFVEFCRNINSTDKSIDTSSLQLYKFQNFQDLSYLSETLISRISSLFTITTI

α13 α14 α15 α16

Nup188 271 LILGLNTSIAQFDIQSPLYMDTETFDTVNSALENDVATNIVNEDPIFHPMIHYSWSFILYYRRALQSSESFDDSDITKFALFAESHDVLQ

α17 α18 α19 α20 α21 α22

Nup188 361 KLNTLSEILSFDPVYTTVITVFLEFSLNFIPITASTSRVFAKIISKAPEQFIENFLTNDTFEKKLSIIKAKLPLLNESLIPLINLALIDT

α23 β1 β2 β3 β4 β5 β6

Nup188 451 EFANFELKDICSFAVTKSSLNDLDYDLIADTITNSSSSSDIIVPDLIELKSDLLVAPPLENENSNCLLSIPKSTKGKILTIKQQQQQQQQ

β7 α24 α25 α26

Nup188 541 QNGQQPPTTSNLIIFLYKFNGWSLVGRILQNLLHSYMEKGTQLDDLQHELMISIIKLVTNVVDPKTSIEKSSEILSYLSNSLDTSASTIN

α27 α28 α29 α30 α31

Nup188 631 GASIIQVIFEIFEISLQRKDYTSIVQCCEFMTMLTPNYLHLVSSYLNKSDLLDKYGKTGLSNMILGSVELSTGDYTFTIQLLKLTKVFIR

α32 α33 α34

Nup188 721 ESLSLKNIHISKRSKIDIINKLILHAIHIFESYYNWKYNNFLQKFEIAFHLTLIFYDVLHDVFTINPHQKDQLIISSSANKLLQLFLTPM

α35 α36 α37 α38 α39

Nup188 811 DSIDLAPNTLTNILISPLNTTTKILGDKILGNLYSKVMNNSFKLCTLLIAIRGSNRDLKPSNLEKLLFINSSKLVDVYTLPSYVHFKVQI

α40 α41 α42 α43

Nup188 901 ELLSYLVEAPWNDDYPFLLSFLGEAKSMAFLKEVLSDLSSPVQDWNLLRSLYIFFTTLLESKQDGLSILFLTGQFASNKKINDESSIDK

α44 α45 α46 α47 α48

Nup188 991 KSSILTVLQKNSLLLDSTPEEVSCKLLETITYVLNTWTNSKIFIKDPKFVNSLLAKLKDSKKLFQKKENLTRDETVSLIKKYKLISRIVE

α49 α50 α51 α52 α53

Nup188 1081 FALCIYNSTDSNSEILNFLNQEDLFELVHHFFQIDGFNKTFHDELNLKFKEKWPSLELQSFQKIPLSRINENENFGYDIPLLDIVLKAD

α54 α55 α56

Nup188 1171 RSWNEPSKSQTNFKEEITDASLNLQYVNYEISTAKAWGALITTFVKRSTVPLNDGFVDLVEHFLKLNIDFGSDKQMFTQIYLERIELSFY

α57 α58 α59 α60

Nup188 1261 LYSFKLSGKLLKEEKIIELMNKIFTIFKSGEIDFIKNIGKSLKNNFYRPLLRSVLVLLELVSSGDRFIELISDQLLEFFELVFSKGVYL

α61 α62 α63

Nup188 1351 LSEILCQINKCSTRGLSTDHTTQIVNLEDNTQDLLLLLSLFKKITNVNPSKNFNVILASSLNEVGTLKVILNLYSSAHLIRINDEPILG

α64 α65 α66 α67 α68 α69

Nup188 1441 QITLTFISELCSIEPIAAKLINSGLYSVLLESPLSVAIQQGDIKPEFSPRLHNIWSNGLLSIVLLLLSQFGIKVLPETCLFVSYFGKQIK

α70 α71 α72 α73

Nup188 1531 STIYNWGDNKLAVSSSLIKETNQLVLLQKMLNLLNYQELFIQPKNSDDQQEAVELVIGLDSEHDKKRLSAALSKFLTHPKYLNSRIIPTT

α74 α75

Nup188 1621 LEEQQQLEDESSRLEFVKGISRDIKALQDSLFKDV

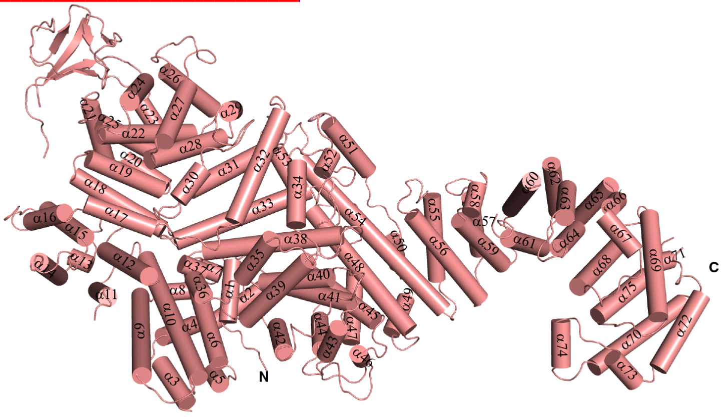

b Nup192

Nup192 1 MKWSAIPFQTLYRSIESGEFDFDLFKEVLPDLQNLNLNTDKLKNNASRSQLEKGEIELSDGSTFKVNQEFIFEAISLSDELNLDEIVACE

Nup192 91 LILSGDTTANNGKVQYFLRRQYILQIVSFIVNCFHEDTELYQELIKNGALVSNILSAFKFIHTQLSEIKQQINKAQILENYNALFQQNIK

Nup192 181 FRRDFLLREYDILSQILYGLVDKGAIMKNKDFILSLLHHVSELDSNDFFIIYYTPAFFHLFASLRVLPDADVKLLHSQFMKDLKDDSIYT

Nup192 271 KPVKVALIFIFFAYFIGWCKEDPKRRADTMDFKTDVDEPMTSAVELGAIEQILIFAADTSIVEQDKSMELFYDIRSLLERHIPRLIPKQL

Nup192 361 LDDEKIFSQTTNSTYNPASATDNMSGRGLWNPSYPGMMSTTGTARLNSMPNNVNEYSYTTIVLSDQTQEFFLSSFDDVLQTIITDCAFLL

Nup192 451 TKIKDAEEDSLLSGEDLTLDDISLKADLERFFLSIYFFYASRPEYSCTFWSDKESNAYGFIEWCSRCNDNLMRSCFYLMVSSLSFGPENA

Nup192 541 LNVYHYFGENSSISWKNIAQCLSDYTKKISNFNSSLHKRQQFSESTHNDIDSTAVALEEGLNEEAVIFLSSLLTLVGSVTYQVDEDVKSS

Nup192 631 LSKVFSDVLFEFTKINTPLVGAAFKVISNLVPKLESSRTKFWSFLDSLIFKDSSLNYSSESYRNAFTNVLTKYSDVLGFLQLFHNLISIH

Nup192 721 SRENNSEYMVFGKLAFPTRLGQGYRKVGIWPYFDYIFNDILAHVDQIVDIRNKRAVQLPILKIIYTGLCSFDYSVILNSIPAAANLDALV

Nup192 811 DCENFFNYVQECPAIPIFNYIFTEKIYKSIFNVVDVGVDQLSIELEGGKNQAELLQLAVKIINKVLDYQETYVEELFPIVKKHGKTDYFL

Nup192 901 PKNYSLHGLRSFYDAIFFNIPLVAHLGLYVGVDDQILATNSLRILAKLSERSNGSVASLSKRNKLLTIFDSVDESARIKDAFITQLESSI

Nup192 991 TDAGVLALKLELLDFLTSNLSNYSRTMTISHLLLGFQVSNVISLGPNLATFISSGTSLLDSLISVLEASLNSITKDNIDYAPMRLATAAL

Nup192 1081 ELKLCRNPLTSGLLYSYLIKENFFERIMILDPQVTRFTTWNGSPFDNSTEEKCKNFIESESVGAFLSFLAYRNYWTQYLGLFIHKISF

Nup192 1171 SGTKSEVLTYVNYLISNTMYSVRLFSFLDPLNYGNICEPKETLSIFTNVPLNLEQVTLNKYCSGNIYDFHKMENLMRLIKRVRAESLHSN

Nup192 1261 SFSLTVSKEQFLKDADVECIKAKSHFTNIISRNKALELNLSVLHSWVQLVQIIVTDGKLEPSTRSNFILEVFGTIIPKISDYIEFNITFS

Nup192 1351 EELVSLAVFLFDIYNRDRKLITDKGTVDGRLYQLFKTCIQGINSPLSSVALRSDFYILANHYLSRVLSDQVGSEKVLQDLRLGSKKLVEI

Nup192 1441 WNDVVYGEGTSRVTGILLLDSLIQLANRSKENFILDSLMKTTRLLLIIRSLKNTDALLNSTTEHINIDDLLYELTAFKATVFFLIRVAE

Nup192 1531 TRGGASALIENNLFRIIAELSFLKVDPDLGLDLMFDEVYVQNSKFLKVNVTLDNPLLVDKDANGVSLFELIVPIFQLISAVLVSMGGSSNK

Nup192 1621 AVVQTVKGLLNTYKRLVIGIFKRDLLREKEDKKNSSDPNNQSLNEMVKLIVMLCTLTGYQNND

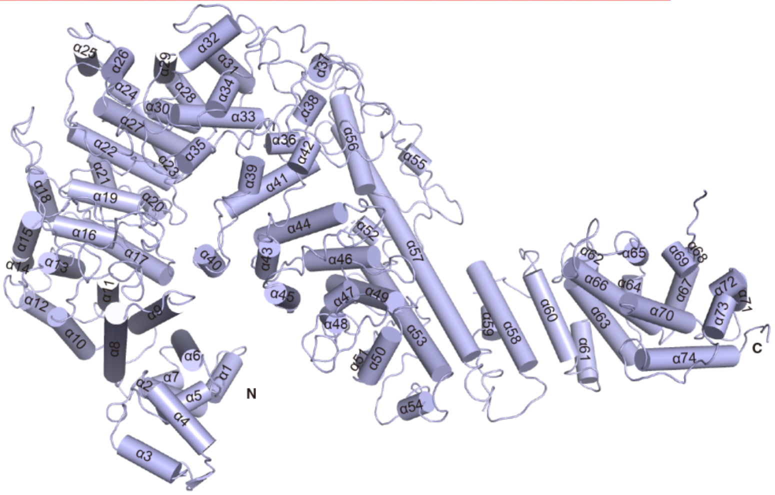

c **Nup157**

α1

Nup157 1 MYSTPLKKRIDYDRETFTASASLGGNRLRNRPRDDQNNGKPNLSSRSFLSERKTRKDVLNKYGEAGNTIESELRDVTTHVKISGLTSSEP

α2 α3 β1 β2 α4 β3 α5 β4

Nup157 91 LQLASEFVQDLSFRDRNTPILDNPDYYSKGLDYNFSDEVGGLGAFTPFQRQQVTNIPDEVLSQVSNTEIKSDMGIFLELNYCWITSDNKL

β5 β6 β7 β8 β9 β10 β11

Nup157 181 ILWNINNSSEYHCIDEIEHTILKVKLVKPSPNTFVSSVENLLIVATLFDIYILTISFNDRTHELNIFNTGLKVNVTGFNVSNIISYERTG

β12 β13 β14 α6 β15 β16

Nup157 271 QIFFTGATDGVNVWELQYNCSENLFNSKSNKICLTKSNLANLLPTKLIPSIPGGKLIQKVLEGDAGTEEETISQLEVDQSRGVLHTLSTK

β17 β18 α7 β19 β20 β21 β22 β23 β24

Nup157 361 SIVRSYLITSNGLVGPVLIDAAHIRRGMNALGVKNSPLLSNRAFKIAKIVSISMCENNDLFLAVITTTGVRLYFKGSISRRSIGSLKLDS

α8 α9 β25 β26 β27

Nup157 451 VKFPPTSISSSLEQNKSFIIGHHPLNTHDTGPLSTQKASSTYINTTCASTIISPGIYFTCVRKRANSGELSKGITNKALLENKEEHKLYV

α10 β28 β29 α11 β30 β31 α12

Nup157 541 SAPDYGILKNYGKYVENTALLDTTDEIKEIVPLTRSFNYTSTPQGYANVFASQYSAEPLKVAVLTSNALEIYCYRTPDEVFESLIENPLP

α13 α14 α15 α16 β32 α17

Nup157 631 FIHSYGLSEACSTALYLACKFNKSEHIKSSALAFFSAGIPGVVEIKPKSSRESGSVPPISQNLFDKSGECDGIVLSPRFYGSALLITRLF

β34 β35 α18 α19

Nup157 721 SQIWEERVFVFKRASKTEKMDAFGISITRPQVEYYLSSISVLADFFNIHRPSFVSFVPPKGSNAITASDAESIAMNALILLINSIKDALS

α20 α21 α22 α23 α24

Nup157 811 LINVFYEDIDAFKSLLNTLMGAGGVYDSKTREYFFDLKFHDLFTPNAKTKQLIKEILIEVVNANIASGTSADYIVNVLKERFGSFCHSAD

α25 α26 α27 α28 α29

Nup157 901 LCYRAGEHLEAAQKFEMIDSKISRNHLDTAIDLYERCAENIELCELRRVVDIMVKLNYQPKTVGFLLRFADKIDKGNQAQEYVSRGCNT

α30 α31 α32 α33

Nup157 991 ADPRKVFYDKRINVYTLIFEIVKSVDDYTSIEQSPSIANISIFSPASSLKKRVYSVIMNSNNRFFHYCFYDWLVANKRQDYLLRLDSQFV

α34 α35 α36 α37 α38

Nup157 1081 LPYLKERAEKSLEISNLLWFYLFKEEHFLEAADVLYALASSDFDLKLSERIECLARANGLCDSSTSFDQKPALVQLSENIHELFDIASIQ

α39 α40 α41 α42

Nup157 1171 DDLLNLVRNETRIDEDYRKQLTLKLNGRVLPLSDLFNDCADPLDYYEIKLRIFKVSQFKDEKVIQGEWNRLLDSMKNAPSPDVGSVGQES

α43 α44 α45 α46 α47

Nup157 1261 FLSSISNTLIRIGKTTRDTDVVFPVHFLMNKILESFIDKSSAADGSVCSMFLLAGVSHLKLYYILSRIIENSEGNVELAKKEMVWLIKDW

α48 α49 α50

Nup157 1351 YQSDSDLRGSIAPEQIKKLEKYDPNTDPVQDYVKDRHHGLK

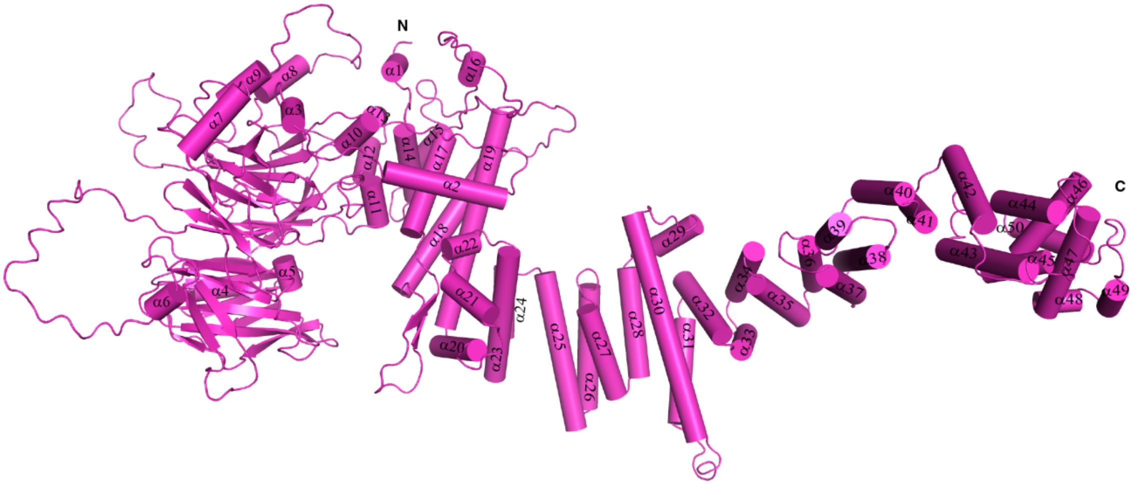

d

Nup170

Nup170 1 MFQSFFHNNGPAAAGETFSDSRSYPLTNHQEVPRNGLNELASSATKAQQQPTHILNSYPITGSNPLMRASAMGATSGSINPNMSNMNEHI

Nup170 91 RVSGMGTSKPLDLAGKYIDHLQHKDSNTPVLDERSYYNSGVDYNFSREKNGLGAFTPFEKQDVFNIPDEILHEFSTSQTKTDMGIFPELN

Nup170 181 RCWITIDNKLILWNINNDNEYQVVDDMKHTIQKVALVRPKPNTFVPAVKHLLLISTTMELFMFAISLDKATNELSVFNTHLSVPVQGIDV

Nup170 271 IDIVSHERSGRIFFAGQASGLNIWELHYSGSDDWFNSKCSKVCLTKSALLSLLPTNMLSQIPGVDFIQALFEDNSNGNGGFSQETITQLT

Nup170 361 DQQRGIIYSLSSKSTIRAYVITEKSLEGPMSIEPAYISRIIGTTTARAAPILGPKYLKIVKISSVAPEENNNLFLVALTVGGVRLYFNG

Nup170 451 SMGRFNIEALRLESIKFPPSSVTPEVIQQELLHQQQEQAKRSFPFFSNLMSSEPVLLKFQKKSSVLLETTKASTIISPGIFFSAVIKSSQ

Nup170 541 QTHQQEKKENSSVTGTTATAGSKTVKQQPVTLQHKLFVSVPDYGILKTHGKYVENATFLETAGPVQQIIPLSGLFNATTKPQGFANEFAT

Nup170 631 QYTSETLRVAVLTSTSIEIYKYRTPDEIFEDLIDNPLPFVLNYGAAEACSTALFVTCKSNKSEKLRSNALTFLTMGIPGVVDIKPVYNRY

Nup170 721 SVSTVSSLLSKPTLSTATTNLQQSITGFSKPSPANKEDFDLDDVILSPRFYGIALLITRLLRDIWGRHVFMTFTDNRVTSHAFISSSDPI

Nup170 811 TPSINNLKSDEISQNRNIISKVSISKDCIEYYLSSINILNEFFITYGDSISQISAPYVLANNSNGRVIDKTEEVANQAESIAINAMIKMV

Nup170 901 QSIKEGLSFLNVLYEESEVEGFDNQYLGFKDIISFVSLDVQKDLVKLDFKDLFAPNDKTKSLIREILLSIINRNITKGASIEYTATALQE

Nup170 991 RCGSFCSASDILGFRAIEHLRRAKEIGLRNYDSLNYHLKNATALLEQIVDDLSIEKLKEAVSMMLSVNYYPKSIEFLLNIANSMDKGKLA

Nup170 1081 CQYVANGFLENDDRKQYYDKRILVYDLVFDTLIKVDELAEKKQSSKTQNQISISNDDEVKLRQKSYEAALKYNDRLFHYHMYDWLVSQNR

Nup170 1171 EEKLLDIETPFILPYLMEKAGSSLKISNILWVYYSRRSKFFESAEILYRLATSNFDITLFERIEFLSRANGFCNSVSPLSQKQRIVQLAS

Nup170 1261 RIQDACEVAGIQGDILSLVYTDARIDSAIKDELIKTLDGKILSTSELFNDFAVPLSYHEIALFIFKIADFRDHEVIMAKWDELFQSLRME

Nup170 1351 FNNTGKKEDSMNFINLLSNVLIKIGKNVQDSEFIFPIFELFPIVCNFFYETLPKEHIVSGSIVSIFITAGVSFNKMYYILKELIETSDSD

Nup170 1441 NSVFNKEMTWLIHEWYKSDRKFRDIISYNDIIHLKEYKIDNDPIEKYVKNSGNNLGICFYKE

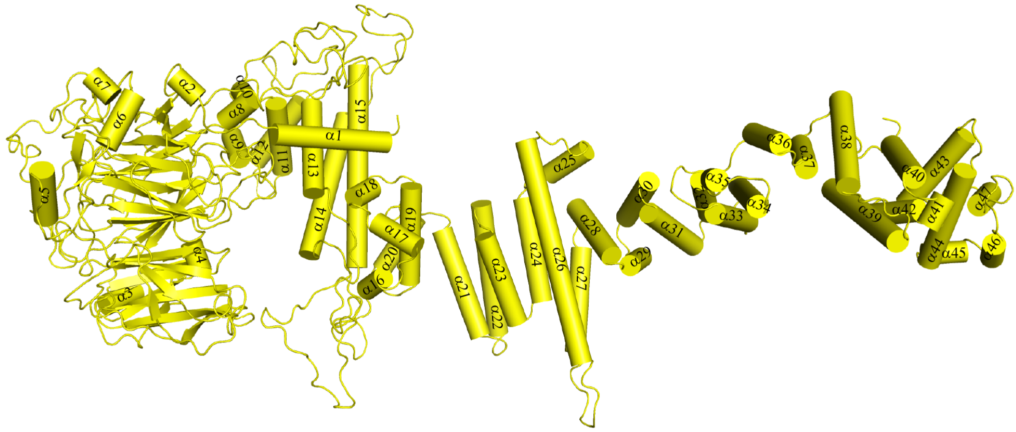

e

Nic96

Nic96 1 MLETLRGNKLHSGTSKGANKKLNELLESSDNLPSASSELGSIQVSINELRRRVFQLRSKNKASKDYTKAHYLLANSGLSFEDVDAFIKDLQT

Nic96 93 NQFLEPNPPKIIESEELEFYIRTKKEENILMSIEQLLNGATKDFDNFINHNLNLDWAQHKNEVMKNFGILIQDKKTVDHKKSISSLDPKLPS

Nic96 185 WGNKGNNILNSNESRLNVNENNILREKFENYARIVFQFNNSRQANGNFDIANEFISILSSANGTRNAQLLESWKILESMKSKDINIVEVGKQ

Nic96 277 YLEQQFLQYTDNLYKKNMNEGLATNVNKIKSFIDTKLKKADKSWKISNLTVINGVPIWALIFYLLRAGLIKEALQVLVENKANIKKVEQSFL

Nic96 369 TYFKAYASSKDHGLPVEYSTKLHTEYNQHIKSSLDGDPYRLAVYKLIGRCDLSRKNIPAVTLSIEDWLWMHLMLIKEKDAENDPVYERYSLE

Nic96 461 DFQNIIISYGPSRFSNYYLQTLLLSGLYGLAIDYTYTFSEMDAVHLAIGLASLKLFKIDSSTRLTKKPKRDIRFANILANYTKSFRYSDPRV

Nic96 553 AVEYLVLITLNEGPTDVELCHEALRELVLETKEFTVLLGKIGRDGARIPGVIEERQPLLHVRDEKEFLHTITEQAARRADEDGRIYDSILLY

Nic96 645 QLAEEYDIVITLVNSLLSDTLSASDLDQPLVGPDDNSETNPVLLARRMASIYFDNAGISRQIHVKNKEICMLLLNISSIRELYFNKQWQETL

Nic96 737 SQMELLDLLPFSDELSARKKAQDFSNLDDNIVKNIPNLLIITLSCISNMIHILNESKYQSSTKGQQIDSLKNVARQCMIYAGMIQYRMPRET

Nic96 829 YSTLINIDVSL

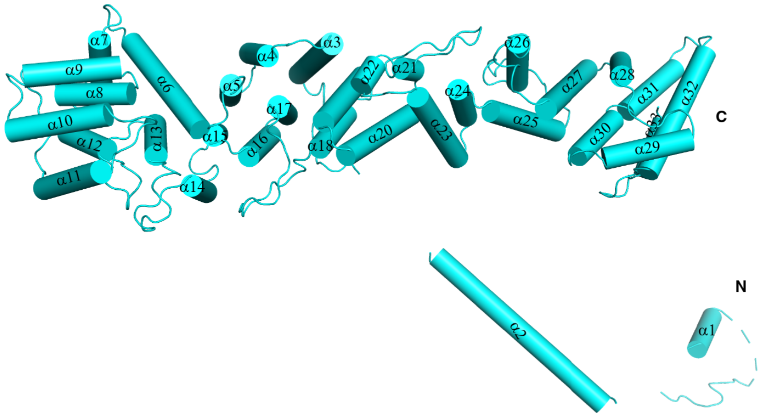

f

Nup57

Nup57 1 MFGFSGSNNGFGNKPAGSTGFSFGQNNNNTNTQPSASGFGFGGSQPNSGTATTGGFGANQATNTFGSNQQSSTGGGLFGNKPALGSLGSSST

Nup57 93 TASGTTATGTGLFGQQTAQPQQSTIGGGLFGNKPTTTTGGLFGNSAQNNSTTSGGLFGNKVGSTGSLMGGNSTQNTSNMNAGGLFGAKPQNT

Nup57 185 TATTGGLFGSKPQGSTTNGGLFGSGTQNNNTLGGGGLFGQSQQPQTNTAPGLGNTVSTQPSFAWSKPSTGSNLQQQQQQIQVPLQQTAQAI

α1 α2

Nup57 277 QQQQLSNYPQQIQEQVLKCKESWDPNTTKTKLRAFVYNKVNETEAILYTKPGHVLQEEWDQAMEKKPSPQTIPIQIYGFEGLNQRNQVQTEN

α3 α4

Nup57 369 VAQARIILNHILEKSTQLQQKHELDTASRILKAQSRNVEIEKRILKLGTQLATLKNRGLPLGIAEEKMWSQFQTLLQRSEDPAGLGKTNELW

α5 α6 α7

Nup57 461 ARLAILKERAKNISSQLDSKLMVFNDDTKNQDSMSKGTGEESNDRINKIVEILTNQQRGITYLNEVLEKDAAIVKKYKNKT

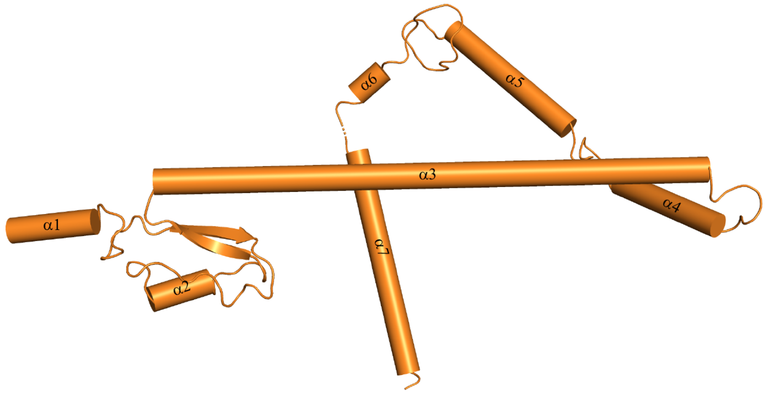

g

Nup49

Nup49 1 MFGLNKASSTPAGGLFGQASGASTGNANTGFSFGGTQTGQNTGPSTGGLFGAKPAGSTGGLGASFGQQQQQSQTNAFGGSATTGGGLFGNKP

Nup49 93 NNTANTGGGLFGANSNSNSGSLFGSNNAQTSRGLFGNNNTNNINNSSSGMNNASAGLFGSKPAGGTSLFGNTSTSSAPAQNQGMFGAKPAGT

Nup49 185 SLFGNNAGNTTTGGGLFGSKPTGATSLFGSSNNNNNNNNSNNIMSASGGLFGNQQQQLQQQPQMQCALQNLSQLPITPMTRISELPPQIRQE

α1

Nup49 277 EQLDQYIQKQVQISHHLKADTIDHDELIDSIPRDVAYLLKSESATSQYLKQDLKKISSFKSLIDEDLLDTQTFSVLLQQLLTPGSKISSND

α2 α3

Nup49 369 LDKFFQKKIHLYEKKLEDYCRILSDIETAVNGIDTDLFGAPNNPNSTAITADLGSSEAENLLQLKTGLAAIVSTVIEEFTLFMDIAERIAVL

Nup49 461 HQKTKTLASLSI

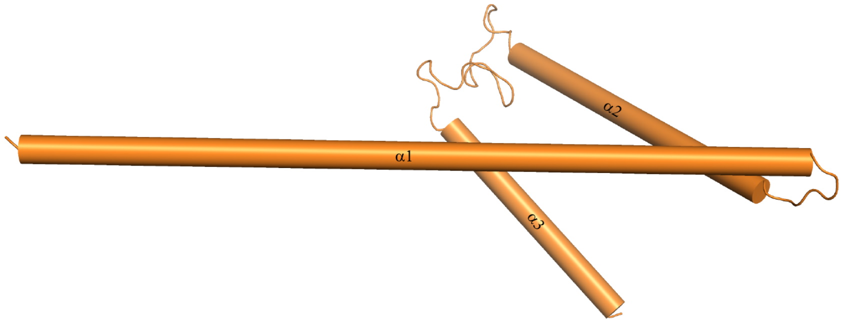

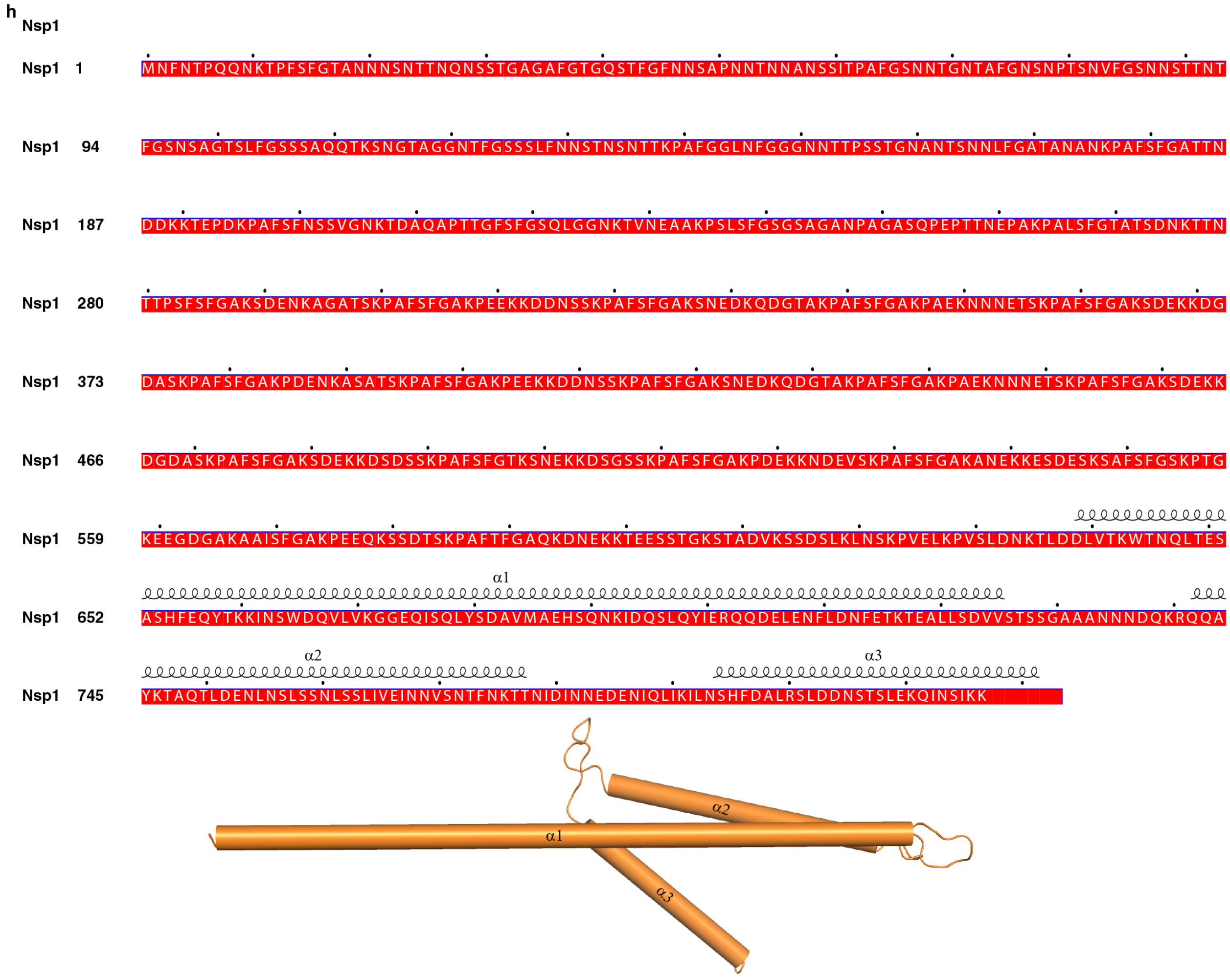

Supplementary information, Fig. S13. Secondary structural information of IR subunits.
(**a-h**) Secondary structural information of Nup188 (a), Nup192 (b), Nup157 (c), Nup170 (d), Nic96 (e), Nup57 (f), Nup49 (g) and Nsp1 (h).
