# Supplementary material for: Near-atomic structure of the inner ring of the Saccharomyces cerevisiae nuclear pore complex
Source: Cell Res. 2022 Mar 18;32(5):437–50. doi: 10.1038/s41422-022-00632-y (PMC9061825; doi:10.1038/s41422-022-00632-y)
Supplement: Supplementary file 14 — Supplementary information, Fig. S14 [file 41422_2022_632_MOESM14_ESM.pdf]

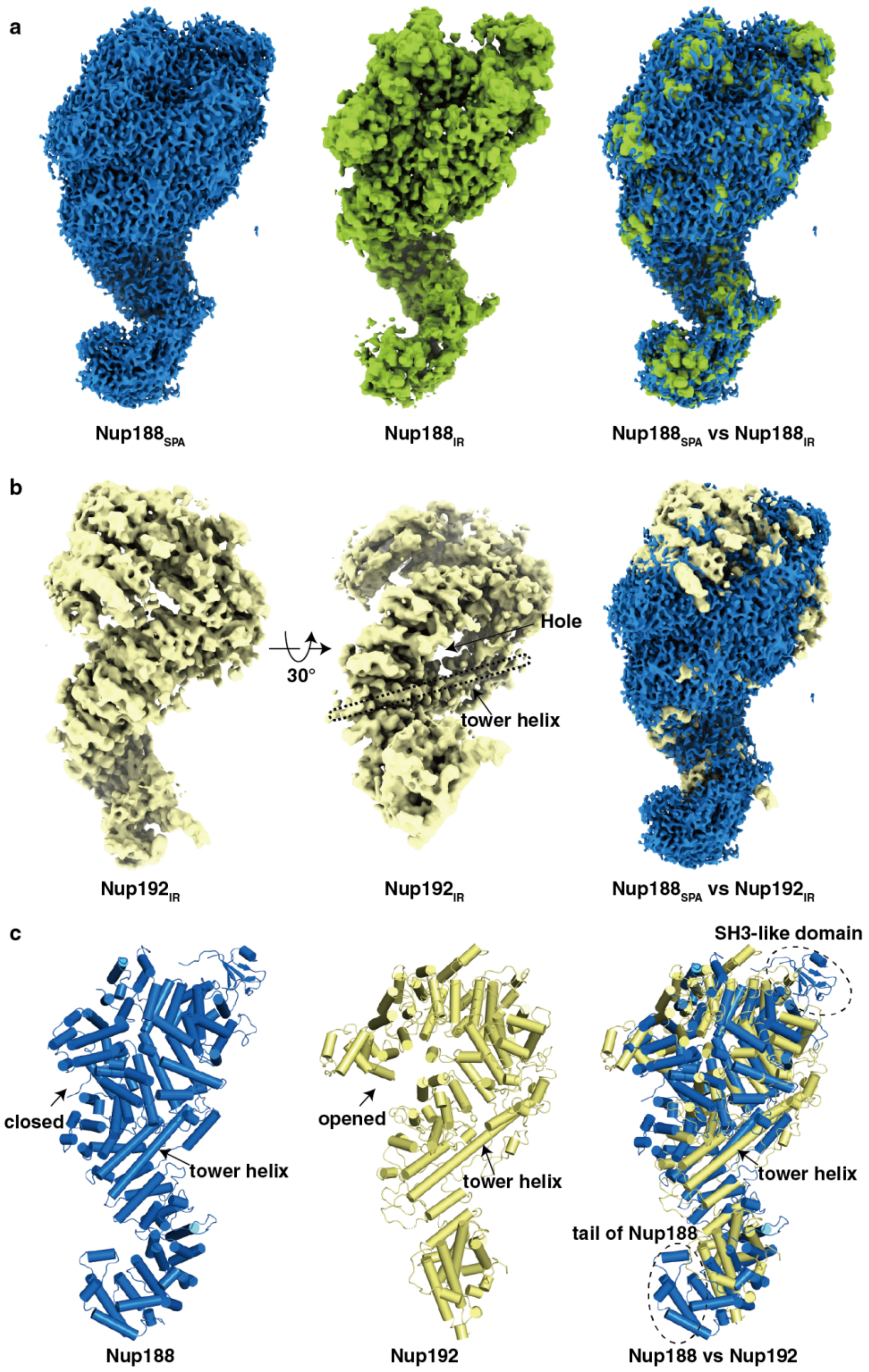

Supplementary information, Fig. S14. Structures of homologous Nup188 and Nup192.
(**a**) Comparison of Nup188 maps from single particle analysis ($Nup188_{SPA}$) and IR monomer map ($Nup188_{IR}$). The superposition of $Nup188_{SPA}$ and $Nup188_{IR}$ is shown in the right panel. (**b**) Comparison of density maps of homologous Nup188 and Nup192. $Nup192_{IR}$ represents Nup192 map from IR monomer map. The superposition of $Nup188_{SPA}$ and $Nup192_{IR}$ is shown in the right panel. (**c**) Comparison of atomic models of Nup188 and Nup192. The Nup188 model is built based on the map of $Nup188_{SPA}$ and Nup192 model is generated by homologous modeling based on crystal structure of Nup192 from *Chaetomium thermophilum* (PDB: 5HB4).
