# Supplementary material for: Near-atomic structure of the inner ring of the Saccharomyces cerevisiae nuclear pore complex
Source: Cell Res. 2022 Mar 18;32(5):437–50. doi: 10.1038/s41422-022-00632-y (PMC9061825; doi:10.1038/s41422-022-00632-y)
Supplement: Supplementary file 15 — Supplementary information, Fig. S15 [file 41422_2022_632_MOESM15_ESM.pdf]

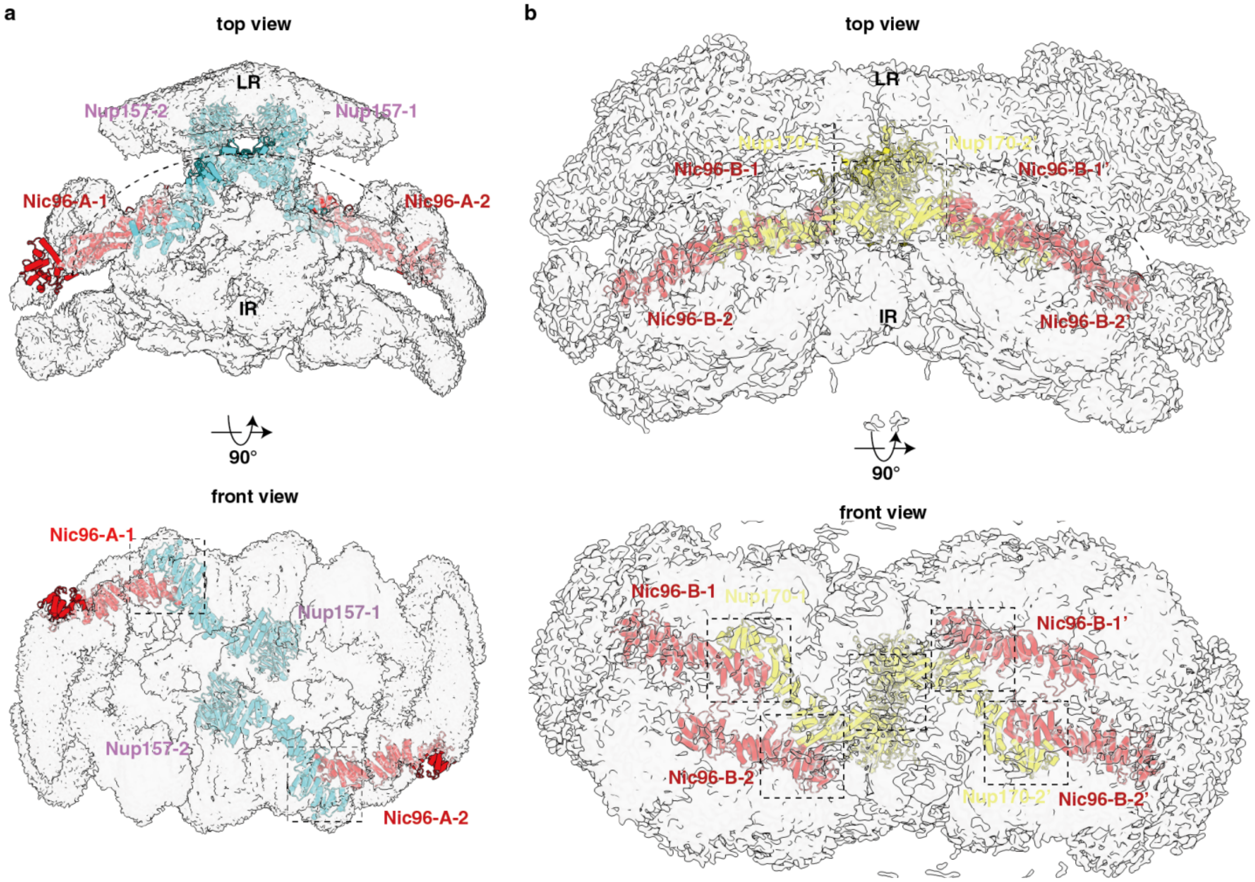

Supplementary information, Fig. S15. Nup157 and Nup170 mediate conjunction between IR and LR.

Nup157 (**a**) and Nup170 (**b**) mediate the conjunction between IR and LR by protruding their N-terminals into LR and C-terminal interactions with Nic96 from IR. Dotted curves indicate boundary of LR and IR. Two views are shown and maps of IR monomer and dimer are shown at a transparency of 70%. Abbreviations: LR, luminal ring; IR, inner ring.
