# Supplementary material for: Near-atomic structure of the inner ring of the Saccharomyces cerevisiae nuclear pore complex
Source: Cell Res. 2022 Mar 18;32(5):437–50. doi: 10.1038/s41422-022-00632-y (PMC9061825; doi:10.1038/s41422-022-00632-y)
Supplement: Supplementary file 16 — Supplementary information, Fig. S16 [file 41422_2022_632_MOESM16_ESM.pdf]

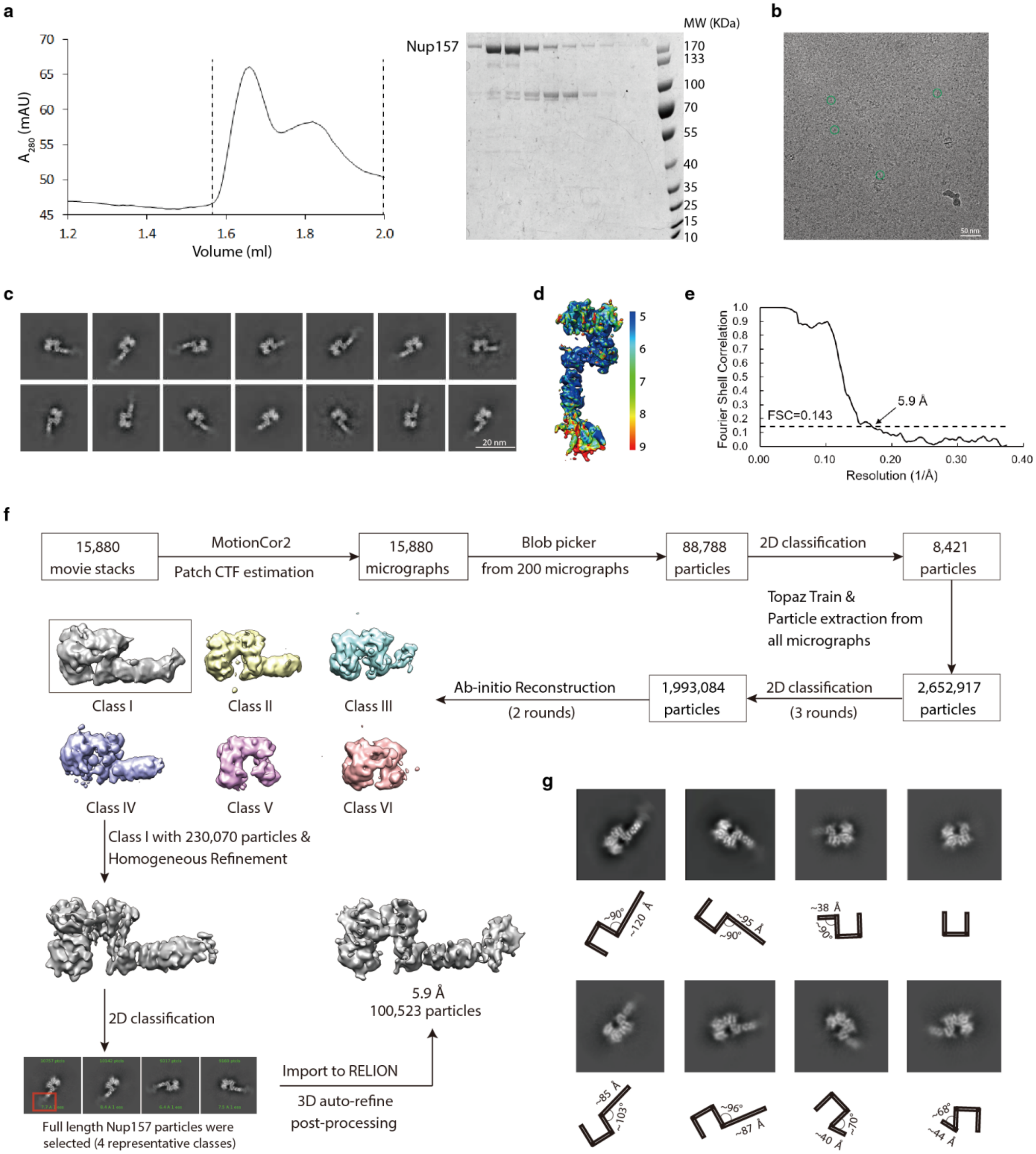

Supplementary information, Fig. S16. Cryo-EM data analysis of Nup157.

(**a**) Purification of Nup157. SEC profile of Nup157 (left panel) and the SDS-PAGE gel of the fractions corresponding to the region between dashed lines on SEC curve (right panel). (**b**) A representative raw cryo-EM image for Nup157 with typical particles marked by green circles. (**c**) Typical good reference-free 2D class averages of Nup157. (**d**) Local resolution of cryo-EM map for Nup157. (**e**) Gold standard FSC curves for the cryo-EM maps of whole Nup157. (**f**) The flowchart for EM data processing and the local resolution maps. Details can be found in "Materials and Methods". (**g**) 2D class average images showing the flexible C-terminal of Nup157.
