# Supplementary material for: Near-atomic structure of the inner ring of the Saccharomyces cerevisiae nuclear pore complex
Source: Cell Res. 2022 Mar 18;32(5):437–50. doi: 10.1038/s41422-022-00632-y (PMC9061825; doi:10.1038/s41422-022-00632-y)
Supplement: Supplementary file 17 — Supplementary information, Fig. S17 [file 41422_2022_632_MOESM17_ESM.pdf]

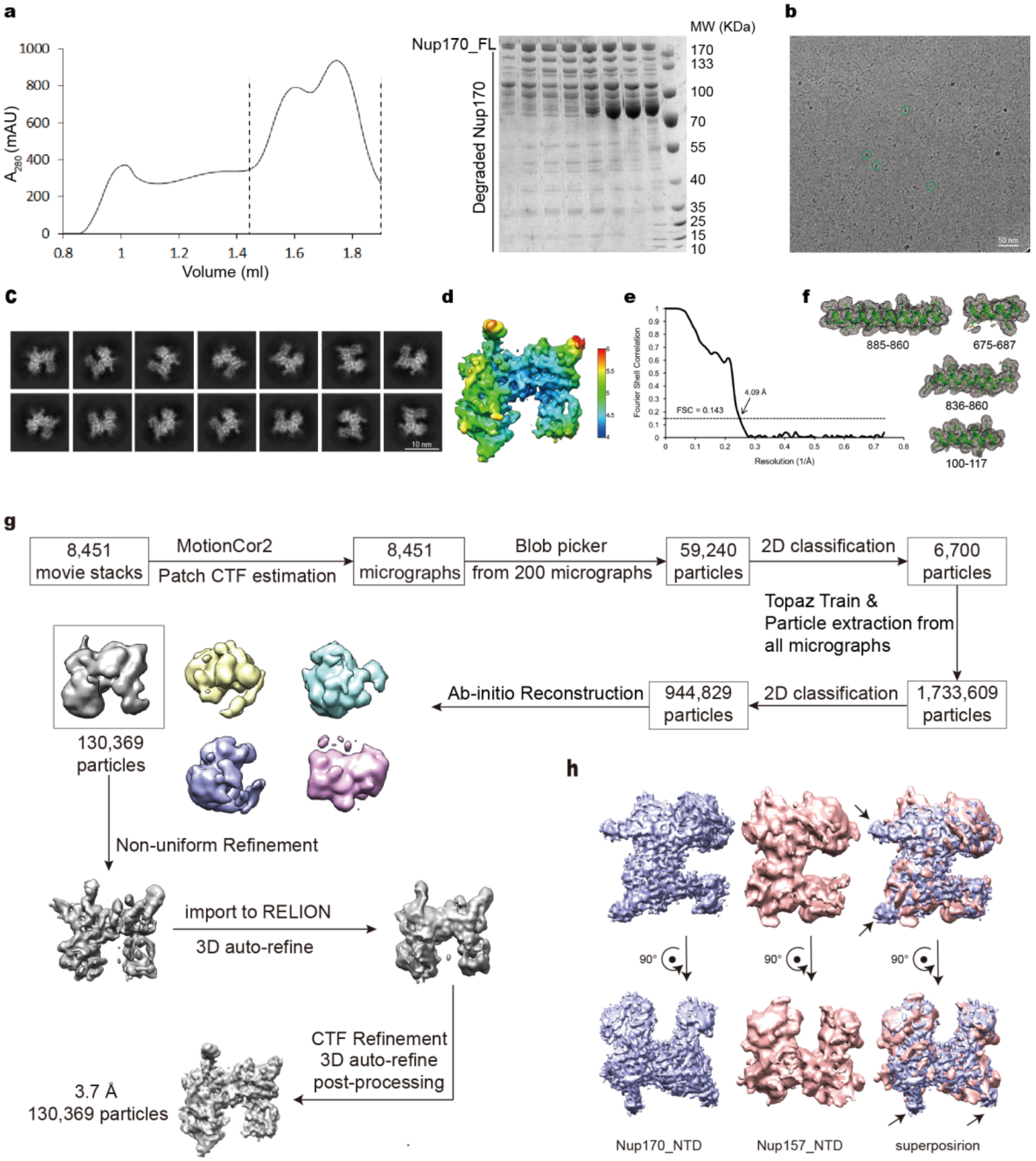

Supplementary information, Fig. S17. Cryo-EM data analysis of Nup170.

(**a**) Purification of Nup170. SEC profile of Nup170 (left panel) and the SDS-PAGE gel of the fractions corresponding to the region between dashed lines on SEC curve (right panel). (**b**) A representative raw cryo-EM image for Nup170 with typical particles marked by green circles. (**c**) Typical good reference-free 2D class averages of Nup170. (**d**) Local resolution of cryo-EM map for Nup170. (**e**) Gold standard FSC curves for the cryo-EM maps of whole Nup170. (**f**) Representative EM density maps for a series of discrete α-helices from the N-terminal of Nup170. (**g**) The flowchart for EM data processing and the local resolution maps. Details can be found in "Materials and Methods". (**h**) Comparison of the density map of the N-terminal domain (NTD) of Nup157 and Nup170. The superposition is shown in the right panel.
