# Supplementary material for: Near-atomic structure of the inner ring of the Saccharomyces cerevisiae nuclear pore complex
Source: Cell Res. 2022 Mar 18;32(5):437–50. doi: 10.1038/s41422-022-00632-y (PMC9061825; doi:10.1038/s41422-022-00632-y)
Supplement: Supplementary file 18 — Supplementary information, Table S1 [file 41422_2022_632_MOESM18_ESM.pdf]

Table S1. Plasmids and yeast strains used in this study.

Plasmids	Expressed strains	Reference
pESC-His-Nup192-Flag	yeast	This study
pESC-Ura-Nup188-Flag	yeast	This study
pESC-Leu-Nup157-Flag	yeast	This study
pESC-Leu-Nup170-Flag	yeast	This study
pGEX-6t-Nup157	BL21(DE3)	This study
pET28at-SUMO-Nup170	BL21(DE3)	This study
pET28at-SUMO-Nup192	BL21(DE3)	This study
pCDNA-Nup157-3xFlag	293F	This study
pCDNA- Nup170-3xFlag	293F	This study
pCDNA- Nup192-3xFlag	293F	This study

Yeast Strains	Genotype	Reference
W303α	*MATα ura3-52 leu2-3,112 his3-11,15 trp1*	Wild type
W303a	*MATa ura3-52 leu2-3,112 his3-11,15 trp1*	Wild type
W303α-Mlp1-PrA	*MATα ura3-52 leu2-3,112 his3-11,15 trp1 Mlp1-TEV-ProteinA::LEU2*	This study
W303α-Nup84-3FH/Mlp1-PrA	*MATα ura3-52 leu2-3,112 his3-11,15 trp1 Nup84-3x Flag-10x His :: URA3 Mlp1-TEV-ProteinA::LEU2*	This study
W303a-Mlp1-PrA	*MATa ura3-52 leu2-3,112 his3-11,15 trp1 Mlp1-TEV-ProteinA::LEU2*	This study
W303a-Nup84-3FH/Mlp1-PrA	*MATa ura3-52 leu2-3,112 his3-11,15 trp1 Nup84-3x Flag-10x His :: URA3 Mlp1-TEV-ProteinA::LEU2*	This study
