# Supplementary material for: Near-atomic structure of the inner ring of the Saccharomyces cerevisiae nuclear pore complex
Source: Cell Res. 2022 Mar 18;32(5):437–50. doi: 10.1038/s41422-022-00632-y (PMC9061825; doi:10.1038/s41422-022-00632-y)
Supplement: Supplementary file 19 — Supplementary information, Table S2 [file 41422_2022_632_MOESM19_ESM.pdf]

Table S2. Cryo-EM data collection and model statistics of NPC and nucleoporins.

	Nup188	IR monomer	IR protomer	IR dimer	IR	NPC	Nup157	Nup170
**Data collection and processing**								
Magnification	130,000	130,000	130,000	130,000	130,000	130,000	130,000	130,000
Voltage (kV)	300	300	300	300	300	300	300	300
Camera	K3	K3	K3	K3	K3	K3	K3	K3
Electron exposure ($e^{-}/Å^2$)	50	50	50	50	50	50	50	50
Defocus range (µm)	-1.5 ~ -2.5	-1.5 ~ -2.5	-1.5 ~ -2.5	-1.5 ~ -2.5	-1.5 ~ -2.5	-1.5 ~ -2.5	-1.5 ~ -2.5	-1.5 ~ -2.5
Pixel size (Å)	0.668	0.668	0.668	0.668	0.668	0.668	0.668	0.668
Micrographs (no.)	16,527	296,820	296,820	296,820	296,820	296,820	15,880	8,451
Initial particle images (no.)	1,208,727	2,238,689	1,266,268	885,259	278,938	279,900	2,652,917	1,733,609
Final particle images (no.)	607,216	633,134	1,266,268	331,211	89,774	51,220	100,523	130,369
Symmetry imposed	C1	C2	C1	C2	C8	C8	C1	C1
Map resolution (Å)	2.8	3.73	3.71	7.69	9.10	12.03	5.9	3.7
Map sharpening B factor ($Å^2$)	-119.6	-143.4	-163.6	-763.5	-860	-1000	-461	-254.4
FSC threshold	0.143	0.143	0.143	0.143	0.143	0.143	0.143	0.143
Map resolution range (Å)	10.0-2.7	10.0-3.7	10.0-3.6	20.0-6.5	30.0-8.0	35.0-12.0	5.0-9.0	3.5-5.5
EMDB number								
**Refinement**								
Initial model used	generated in RELION3.1							
Model composition								
Non-hydrogen atoms	12,743	139520	69760					
Protein residues	1,581	17410	8705					
R.m.s. deviations								
Bond lengths (Å)	0.002	0.017	0.017					
Bond angles (°)	0.489	1.674	1.674					
Validation								
MolProbity score	1.59	3.05	3.02					
Clashscore	5.55	35.01	32.70					
Rotamer outliers (%)	0	4.01	4.00					
Cβ outliers (%)	0	0.88	0.88					
Ramachandran plot								
Favored (%)	95.86	89.54	89.52					
Allowed (%)	4.14	9.22	9.25					
Disallowed (%)	0	1.23	1.23					
PDB accession number								
