# Supplementary material for: Near-atomic structure of the inner ring of the Saccharomyces cerevisiae nuclear pore complex
Source: Cell Res. 2022 Mar 18;32(5):437–50. doi: 10.1038/s41422-022-00632-y (PMC9061825; doi:10.1038/s41422-022-00632-y)
Supplement: Supplementary file 20 — Supplementary information, Video legend [file 41422_2022_632_MOESM20_ESM.pdf]

Supplementary information, Video S1. The motion of flexible C-terminal region of Nup188 indicated by the 2D class average image obtained by EMAN2.

Supplementary information, Video S2. The motion of flexible C-terminal region of Nup188 indicated by the 3D density map obtained by RELION.

Supplementary information, Video S3. The motion of flexible C-terminal region of Nup157 indicated by the 2D class average image obtained by EMAN2.

Supplementary information, Video S4. The molecular architecture of the IR monomer.
